# Transcriptional Analysis of PRRSV-Infected Porcine Dendritic Cell Response to *Streptococcus suis* Infection Reveals Up-Regulation of Inflammatory-Related Genes Expression

**DOI:** 10.1371/journal.pone.0156019

**Published:** 2016-05-23

**Authors:** Gaël Auray, Claude Lachance, Yingchao Wang, Carl A. Gagnon, Mariela Segura, Marcelo Gottschalk

**Affiliations:** Groupe de recherche sur les maladies infectieuses en production animale (GREMIP), Faculty of Veterinary Medicine, University of Montreal, 3200 Sicotte, St-Hyacinthe, Québec, Canada, J2S 2M2; University of Lleida, SPAIN

## Abstract

The porcine reproductive and respiratory syndrome virus (PRRSV) is one of the most important swine pathogens and often serves as an entry door for other viral or bacterial pathogens, of which *Streptococcus suis* is one of the most common. Pre-infection with PRRSV leads to exacerbated disease caused by *S*. *suis* infection. Very few studies have assessed the immunological mechanisms underlying this higher susceptibility. Since antigen presenting cells play a major role in the initiation of the immune response, the *in vitro* transcriptional response of bone marrow-derived dendritic cells (BMDCs) and monocytes in the context of PRRSV and *S*. *suis* co-infection was investigated. BMDCs were found to be more permissive than monocytes to PRRSV infection; *S*. *suis* phagocytosis by PRRSV-infected BMDCs was found to be impaired, whereas no effect was found on bacterial intracellular survival. Transcription profile analysis, with a major focus on inflammatory genes, following *S*. *suis* infection, with and without pre-infection with PRRSV, was then performed. While PRRSV pre-infection had little effect on monocytes response to *S*. *suis* infection, a significant expression of several pro-inflammatory molecules was observed in BMDCs pre-infected with PRRSV after a subsequent infection with *S*. *suis*. While an additive effect could be observed for CCL4, CCL14, CCL20, and IL-15, a distinct synergistic up-regulatory effect was observed for IL-6, CCL5 and TNF-α after co-infection. This increased pro-inflammatory response by DCs could participate in the exacerbation of the disease observed during PRRSV and *S*. *suis* co-infection.

## Introduction

The porcine reproductive and respiratory syndrome (PRRS), caused by a RNA virus (PRRSV), is characterized by weak live-born pigs, severe pneumonia, reduction in growth performances, mortality, and abortions [1]. At least two genotypes of PRRSV can be distinguished: PRRSV type 1 (with different sub-types), which comprises strains from Europe; and PRRSV type 2, which includes strains from North America [2]. This disease has become the most prevalent in pigs worldwide and is responsible for great economic losses [3]. It has been proposed that the immune response during PRRSV infection is characterized by an altered innate immune response and a weak and delayed adaptive immune response [4].

*Streptococcus suis* serotype 2 is an important swine bacterial pathogen responsible for meningitis, septicemia with sudden death and other infections in pigs. It is also an emerging zoonotic agent [5–7]. *S*. *suis* usually infects pigs via the respiratory tract by colonizing the palatine and nasopharyngeal tonsils, potentially disseminating to the draining lymph nodes of the upper respiratory tract [8]. By mechanisms not well understood, virulent strains of *S*. *suis* will cross the first natural lines of the host defense and enter the bloodstream to initiate disease. Among the described potential virulence factors [9], the capsular polysaccharide (CPS) displays anti-phagocytic properties and interferes with internalization of *S*. *suis* by dendritic cells (DCs), monocytes/macrophages and neutrophils [10–13]. Interestingly, some studies originally suggested that *S*. *suis* may eventually be internalized by phagocytes and travel intracellularly in the bloodstream (Trojan Horse theory) [14]. The pathology caused by *S*. *suis* has been mainly associated with an excessive inflammatory response [15,16], through the production of pro-inflammatory cytokines and chemokines by cells of the immune system such as monocytes/macrophages [17,18] and DCs [11].

Previous studies have shown increased severity of disease in animals where PRRSV was found in co-infection with a wide range of pathogens [19–24], while others were controversial [25,26]. More precisely, PRRSV and *S*. *suis* co-infection cases are frequently observed in the field, but relatively few scientific studies have yet documented this interaction. In *S*. *suis*-infected piglets, pre-infection with PRRSV has been shown to induce a higher mortality rate compared to those infected by either PRRSV or *S*. *suis* alone [24,27,28]. An enhanced bacteremia and bacterial tissue colonization of co-infected animals was also observed, with both PRRSV and *S*. *suis* found in the bloodstream [24,29]. Piglets born from PRRSV-infected gestating sows were also shown to be more susceptible to a subsequent *S*. *suis* infection [30]. Although it has been suggested that the higher susceptibility of co-infected animals might be due to an immunomodulation induced by the PRRSV infection, the exact mechanisms involved in such interactions are still unknown [28].

Monocytes/macrophages and DCs are antigen presenting cells (APCs) that take up pathogens, process these, and present pathogen-derived antigens to other cells of the adaptive immune system. They are both important mediators of the innate immune response which recognize conserved pathogen-associated molecular patterns (PAMPs) through their expression of pattern recognition receptors (PRRs). While monocytes are involved in the inflammatory response to infection, DCs are professional APCs and are key cells that can shape the adaptive immune response as they provide a link between innate and adaptive immunity [31,32].

In this study, the *in vitro* response of PRRSV-infected porcine DCs and monocytes to a further *S*. *suis* infection was investigated. The effect of PRRSV on the phagocytosis and intracellular survival of *S*. *suis* by both cell populations was first assessed. A genomic approach was then used to compare the gene expression profiles, mainly those involved in inflammation, of both cell types infected with *S*. *suis*, with or without a previous infection with PRRSV.

## Materials and Methods

### Animals

Six week-old Dutch Landrace pigs were purchased from a high health status herd, which was shown to be negative for PRRSV and other major porcine respiratory diseases, including enzootic pneumonia, swine pleuropneumonia and porcine circovirus-associated diseases. This herd did not show any enzootic episode of acute disease due to *S*. *suis* for at least the last three years. All animal experiments were conducted in accordance with the ethical guidelines and policies of the Canadian Council of Animal Care and specifically approved by the Animal Welfare Committee of the University of Montreal (certificate number RECH-1570).

### *S*. *suis* and PRRSV strains and culture conditions

The wild-type virulent *S*. *suis* serotype 2 strain P1/7 has been widely used in previous studies [33]. In some experiments, and for comparison purposes, its isogenic non-encapsulated mutant, Δ*cps2f*, was also used [34]. Bacteria were grown overnight on Todd-Hewitt agar (Becton Dickinson, Sparks, MD, USA) or sheep blood agar plates at 37°C. Three colonies were then isolated and cultured for 8 h in Todd-Hewitt broth (THB, Becton Dickinson) at 37°C without agitation. Ten μl of a 1/1,000 dilution of the 8 h cultures were then transferred into 30 ml of THB and incubated at 37°C for another 16 h. Bacteria were washed twice in phosphate-buffered saline (PBS; pH 7.3). The bacterial pellet was resuspended and adjusted to a concentration of 5 x 10^8^ Colony Forming Units (CFU)/ml, as determined by plating samples onto THA using an Autoplate® 4000 Automated Spiral Plater (Spiral Biotech, Norwood, MA).

The IAF-Klop PRRSV genotype 2 strain, previously isolated in Canada, was used [35]. The PRRSV virus stock was obtained by freezing and thawing MA104-derived monkey kidney (MARC-145) cells infected with the PRRSV. Virus was then purified by ultracentrifugation (3.5 h at 83,000 x *g*) on a 30% sucrose gradient in Tris-buffered saline (pH 7.5). Virus pellets were resuspended in PBS and frozen at -70°C for further use. The virus stock was titrated by assessing the 50% tissue infectious dose (TCID_50_) in MARC-145 cells as previously described [36]. Titres of 10^7.5^ TCID_50_/ml were obtained for the virus stocks used in this study.

### Monocyte isolation and BMDC generation

For monocyte isolation, pig blood from four different animals was collected in 10 ml EDTA-coated vacutainers and independently processed (BD Bioscience, Mississauga, ON, Canada). The vacutainers were centrifuged for 30 min at 400 x *g* and the buffy coat resuspended in 7.5% PBS-EDTA and layered on a 60% FICOLL-PAQUE® Plus gradient (GE Healthcare Bio-Sciences, AB, Uppsala, Sweden) to isolate peripheral blood mononuclear cells (PBMCs). Monocytes were further isolated as described elsewhere [37]. Briefly, PBMCs were incubated for 20 min with anti-human CD14 microbeads (Miltenyi Biotec Inc., Auburn, CA), and CD14^+^ monocytes were positively selected using the Magnetic Activated Cell Sorting system (MACS®; Miltenyi) and plated in 24-well plates (Fisher Scientific, Pittsburgh, PA).

Bone marrow-derived DCs (BMDCs) were generated as previously described [11]. Briefly, hematopoietic cells were isolated from the femur bone marrow of four different animals and independently processed. Following erythrocyte lysis, cells were cultured in complete RPMI-1640 medium supplemented with 10% heat-inactivated fetal bovine serum, 2 mM L-Glutamine, 10 mM HEPES, and 100 U/ml Penicillin-Streptomycin (all reagents from Invitrogen, Life Technologies Inc., Burlington, ON, Canada). This complete medium was complemented with 100 ng/ml of porcine recombinant GM-CSF (rpGM-CSF; Cell Sciences, Canton, MA, USA). Cells (5 x 10^5^ cells/ml) were cultured in 10 ml of complete medium at 37°C in a 5% CO_2_ incubator. After 3 days of culture, 10 ml of fresh complete medium with rpGM-CSF was added, and at day 6, half of the medium was removed and replaced with fresh complete medium containing rpGM-CSF. Cells were harvested after 8 days of culture and plated in 24-well plates (Fisher Scientific). Cells were characterized and confirmed as DCs as previously described [11].

### *In vitro* PRRSV infection, *S*. *suis* infection and co-infection

Cells were plated at 1 x 10^6^ cells/ml in 1 ml of complete RPMI and rested for 3 h before use. For PRRSV infection, medium was removed and replaced with 300 μl of serum-free complete RPMI with PRRSV IAF-Klop (MOI of 0.1) for 2 h. The wells were washed twice with PBS to remove free virus and 1 ml of complete RPMI was added to each well. The amount of PRRSV genome RNA was determined at different incubation times in both blood monocytes and BMDCs, using a RT-qPCR assay as previously described [38]. The QIAamp Viral RNA kit (Qiagen) was used to isolate viral RNA from PRRSV-infected cell lysates according to the manufacturer’s instructions. A commercial PRRSV RT-qPCR diagnostic kit (NextGen, Tetracore Inc., Gaithersburg, MD, USA) was used for PRRSV quantification as recommended by the manufacturer. The PRRSV RT-qPCR results were expressed in cycle threshold (Ct).

For co-infection experiments, cells were infected with PRRSV as described above. After 2 h of infection, cells were washed with PBS. *S*. *suis* strain P1/7 was then added at a MOI of 1 (1 x 10^6^ CFU) for the phagocytosis assays or at a MOI of 0.1 (1 x 10^5^ CFU) for the microarray assay. Bacteria were left in culture with the cells from 30 min to 5 h for phagocytosis and intracellular survival assays, respectively, and 12 h for the microarray assay. A lower bacterial MOI was used for microarray assays since otherwise toxicity killed more than 75% of cells at 12 h of incubation (results not shown). In parallel studies, virus-infected cells were left without bacterial infection (virus infection alone) or were virus-mock infected, similarly incubated and then infected with *S*. *suis* (bacterial infection alone). Finally, virus- and bacterial-mock infected cells were used as negative controls. Under the conditions used, low cell toxicity levels were confirmed for all conditions using Cytotox 96 kit (Promega, Madison, WI) from culture supernatants according to manufacturer’s instructions.

### Phagocytosis assay and intracellular survival

Bacterial phagocytosis and intracellular survival assays were performed as previously described with some modifications [11]. Briefly, *S*. *suis* P1/7 (MOI:1) was added to each well and phagocytosis was left to proceed for either 30 min, 60 min or 90 min. Incubation time was chosen based on our own previous published results [11]. For co-infections, cells were pre-infected with PRRSV IAF-Klop as described in the previous section. For each incubation time, extracellular bacteria were killed by adding penicillin G (5 μg/ml) and gentamicin (100 μg/ml) (Sigma, Oakville, ON, Canada) for 1 h. Cells were then washed three times with PBS and the last wash kept as a control to ensure all extracellular bacteria were killed. One ml of sterile water was then added to each well in order to lyse the cells. The number of viable intracellular bacteria was assessed by plating serial dilutions of the lysates on THA plates. The number of CFU/ml in the final suspension was determined by plating samples onto THA as described above. To study intracellular survival, bacteria were left for 60 min to be internalized by the cells. As described above, a penicillin G and gentamicin solution was added but this time, cells were cultured for an additional 1 h, 3 h or 5 h in the presence of antibiotics. Cells were then processed as described above and the surviving intracellular bacteria counted.

### Cell activation for microarray studies: homogenization and extraction of total RNA from monocytes and BMDCs

12 h following bacterial infection, supernatants were removed from the wells of infected and control cells and 600 μl of lysis buffer (Qiagen, Mississauga, ON, Canada) was added. Incubation time was chosen based on preliminary data of the kinetics of eight pro-inflammatory genes evaluated by qRT-PCR (data not shown). Shorter incubation times showed low and inconsistent results and higher incubation times led to high toxicity. Total RNA was isolated using an RNeasy minikit with on-column DNase digestion (Qiagen). RNA extraction was performed as per the manufacturer’s instructions and each RNA sample was divided into two aliquots (one for microarray, the other for qPCR) that were kept at -80°C until use.

### Agilent microarray analysis

RNA samples were sent to the McGill University and Genome Quebec Innovation Centre (Montréal, Québec, Canada) for microarray analysis using the Agilent porcine (v2) gene expression microarray 4x44K (Agilent Technologies, Santa Clara, CA). Before performing the microarray analysis, RNA sample quality and quantity were assessed using an Agilent 2100 bioanalyser (Agilent Technologies). The microarray was performed as per the manufacturer’s instructions and the samples were randomly distributed on the chips.

### Microarray data accession number

All raw microarray data are available and have been deposited on the Gene Omnibus Expression database under accession numbers GSE75687.

### Validation of microarray data by real-time RT-qPCR

In order to validate the microarray data, 10 genes from DCs were selected and RT-qPCR analysis was performed (Table 1). Three of these genes were also tested in activated monocytes. These experiments were run and analyzed in accordance with the minimum information for publication of quantitative real-time qPCR experiment (MIQE) guidelines [39]. The validation of the microarray data was performed with another aliquot of the same RNA sample used for the microarray experiments. The cDNA was generated by reverse transcription of 500 ng of total RNA using the Quantitect cDNA synthesis kit (Qiagen) as per the manufacturer’s instructions. The real-time qPCR experiments were performed on a CFX-96 rapid thermal cycler system (Bio-Rad, Hercules, CA) using gene-specific primers (250 nM) and the SsoFast Evagreen supermix kit (Bio-Rad), as per the manufacturer’s instructions. The cycling conditions were 3 min of polymerase activation at 98°C, followed by 40 cycles at 98°C for 2 s and 57°C for 5 s. Melting curves were generated after each run to confirm the presence of a single PCR product. The sequences of the primers (Integrated DNA technologies, Coralville, IA) used for qPCR are shown in Table 1 and were verified to have reaction efficiencies between 90% and 110%. The housekeeping genes GAPDH and Hypoxanthine were found to be the most stably expressed (data not shown) using the GeNorm applet v.3.5 (**http://medgen.ugent.be/~jvdesomp/genorm/**) and were therefore used as reference genes to normalize the data. Fold changes in gene expression were calculated using the comparative cycle threshold (Ct) method [40] using the CFX software manager v.3.0 (Bio-Rad). Samples from mock-infected monocytes or BMDCs were used as calibrators.

**Table 1:** Primer sequences used for real-time qPCR assays.

Gene	Genbank accession no. /Reference	Amplicon size (bp)	Forward (F) and Reverse (R) primers
***Il6***	NM_214399	105	F: ACTCCCTCTCCACAAGCGCCTT R: TGGCATCTTCTTCCAGGCGTCCC
***Cxcl8***	NW_003300390	80	F: TGTGAGGCTGCAGTTCTGGCAAG R: GGGTGGAAAGGTGTGGAATGCGT
***Tnf***	NM_214022	112	F: GCCACCACGCTCTTCTGCCTA R: ACGATGATCTGAGTCCTTGGGCCA
***Il12p40***	NM_214013	148	F: CTGAAGAAGACGGCATCACG R: AGGAGTGACTGGCTCAGAAC
***Il23p19***	NM_001130236	82	F: CTCCTTCTCCGCCTCAAGATCC R: TTGCTGCTCCATGGGCGAAGAC
***Il15***	NM_214390	164	F: CTGAGGATGGCATTCATGTC R: GGGATGAGCATCACTTTCAG
***Ifnb***	NM_001003923	150	F: TGCAACCACCACAATTCCAGAAGG R: TCTGCCCATCAAGTTCCACAAGGA
***Ccl20***	NM_001024589	147	F: TGCTCCTGGCTGCTTTGATGTC R:TCATTGGCGAGCTGCTGTGTG
***Cox2***	NM_214321	165	F: TAGGATTCAGGGCTTTCACTGGCT R: TGTCAGCCGACAATGAGATGTGGA
***Nod2***	[41]	66	F: GAGCGCATCCTCTTAACTTTC R: ACGCTCGTGATCCGTGAAC
***Gapdh***	XM_003126534	87	F: CTGAGACTCAACGAAGCTCACTGGC R: TTCTCCAGGCGGCAGGTCAGA
***Hypox***	NM_001032376	142	F: GCAGCCCCAGCGTCGTGATT R: CGAGCAAGCCGTTCAGTCCTGT

### Statistical analysis

For PRRSV infectivity assays, a two-way analysis of variance (ANOVA) assay was performed to compare viral replication in monocytes and BMDCs. For phagocytosis assays and intracellular survival assays, data are presented as the mean ± standard error of the mean. A Wilcoxon matched-pairs ranked test was performed to compare the groups or One-way ANOVA followed by Student-Newman-Keuls secondary analysis were performed to evaluate statistical differences between BMDCs and monocytes. Prism v.5 (Graphpad) was used for data analysis. *P* < 0.05 was considered statistically significant.

For microarray data analysis, text files containing the signal and detection *P*-values per probe for each sample were imported into FlexArray software v.1.6.2 (McGill University and Genome Quebec Innovation Centre; http://gqinnovationcenter.com/services/bioinformatics/flexarray/index.aspx?l=e). Data were first processed by analyzing box plot of sample intensity to ensure that signal intensity data were comparable between samples. Data were then processed by applying a variance-stabilizing normalization (VSN) filter in order to normalize the datasets. The VSN method fits the VSN model to raw microarray data. In contrast with other methods of preprocessing two-color microarray data, VSN normalization is a one-step procedure. The data are returned on a generalized logarithm scale to base 2. A principal component of analysis plot was created to observe separation of different treatment groups. No outliers were removed from the data. Scatter plots of expression were then analyzed to ensure that probe data within a treatment group were not differentially expressed but also to verify that some probes were differentially expressed between different treatment groups, indicating possible differentially expressed genes. Afterwards, ANOVA was used to search for differentially expressed genes between infected and mock-infected groups. ANOVA results were then post-processed in flexarray by a FDR correction using a Benjamini Hochberg algorithm. Differentially expressed genes were defined by fold changes greater than 2-folds or smaller than 0.5 fold with an accompanying *P*-value ≤ 0.05. Heatmap was generated using VSN normalized data (log_2_ based) generated from FlexArray software v.1.6.2. This data was then processed using Gene Cluster 3.0 (Stanford University) [42]. A hierarchical clustering data set was generated using a complete linkage method with uncentered correlation as similarity measure. The clustering data generated by Gene Cluster 3.0 was then visualized using Java Tree View V 1.1.6r4 (Stanford University) [43].

For RT-qPCR analysis, fold-changes of gene expression were calculated using the CFX software manager v.3.0 (Bio-Rad). Samples from mock-infected cells were used as calibrator. Results were analyzed using Sigmaplot 12.5 (Systat, Chicago, IL), and ANOVA was performed to measure statistical differences between groups. Differences were considered statistically significant at *P* ≤ 0.05. A Pearson correlation analysis was done to evaluate correlation of RT-qPCR data to microarray study for the validation of the microarray data.

## Results

### BMDCs are more susceptible than monocytes to PRRSV infection

**Fig 1:**
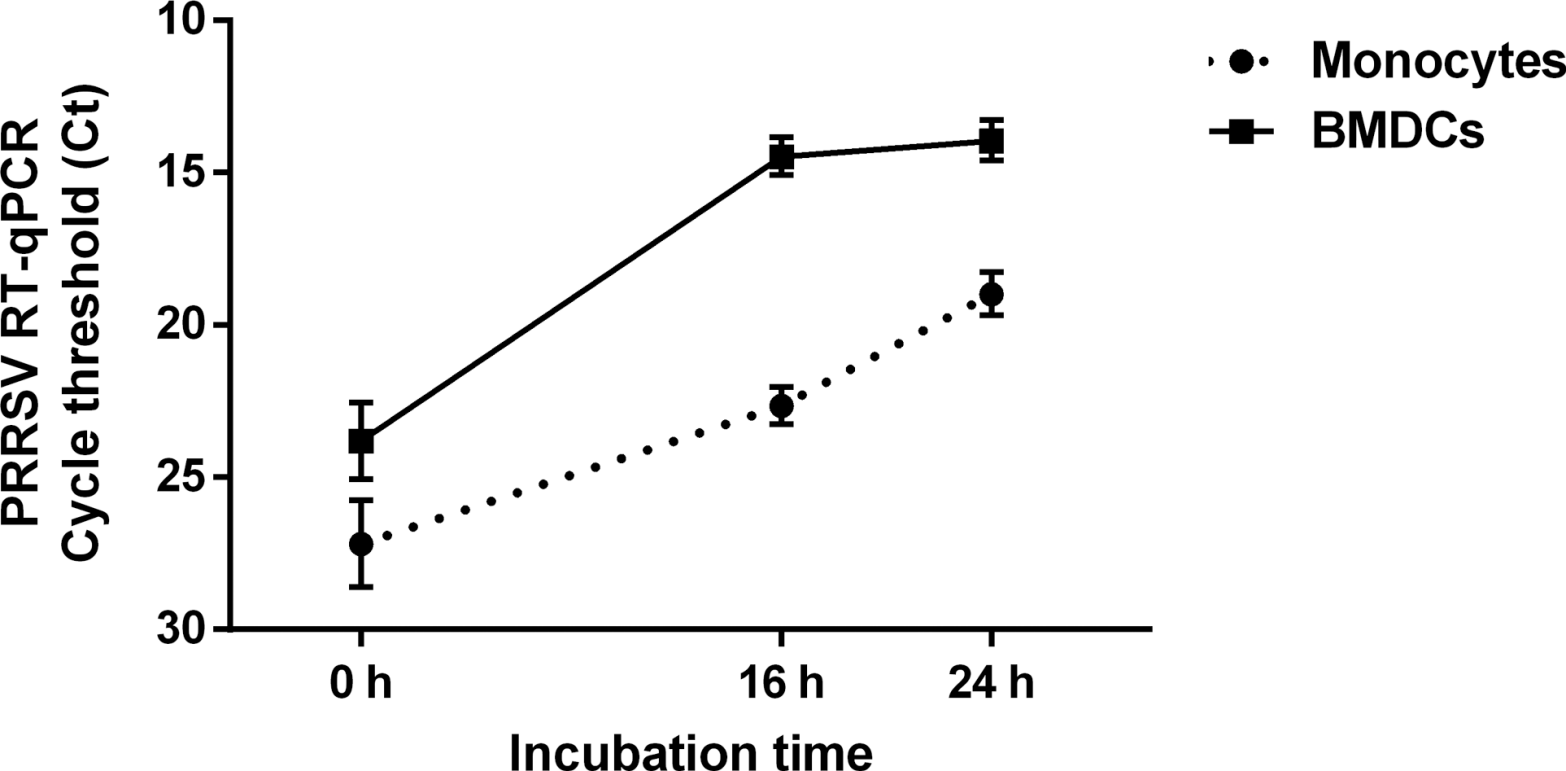
PRRSV quantification in porcine bone marrow-derived dendritic cells (BMDCs) and porcine monocytes. Porcine BMDCs and porcine monocytes were cultured for 2 h in presence of PRRSV virus before washing the cells to remove extracellular virus (t = 0 h). Cells were then cultured for an additional 16 h and 24 h and PRRSV genome RNA was determined using RT-qPCR and expressed in cycle threshold (Ct).

Before studying the co-infection between PRRSV and *S*. *suis*, the toxicity as well as the infectivity and the replication of the IAF-Klop PRRSV and *S*. *suis* strains when cultured with porcine monocytes and BMDCs was assessed. Preliminary studies with different bacterial/virus ratios and incubation times were tested and those used in the present study represent the best combination that avoided toxicity. In fact, higher virus or bacterial concentration resulted in high toxicity for cells (data not shown). PRRSV replicated in both cell types, significantly increasing the amount of viral RNAs with time (Fig 1). Following PRRSV infection, viral replication in BMDCs is significantly higher than that in monocytes. BMDCs are more permissive to the virus (*P* < 0.05), since at t = 0 h (2 h after incubation with virus), PRRSV load was already greater in BMDCs (23.8 ± 1.3 Ct) compared to monocytes (27.2 ± 1.4 Ct). The virus also replicated more rapidly in BMDCs (*P* < 0.05). Indeed, after 16 h of infection, the viral load in BMDCs reached 14.5 ± 0.6 Ct, compared with 22.7 ± 0.6 Ct in monocytes. The RT-qPCR values corresponded to an increase from 10^2^ at 0 h to almost 10^5^ TCID_50_/ml in BMDCs after 16 of infection (data not shown). Between 16 h and 24 h of culture, PRRSV replication reached a plateau in BMDCs, whereas for monocytes, a delayed but continuous viral replication was observed after 16 h; viral load was nonetheless still lower than that of BMDCs. Bacterial replication was also highly significant, increasing from originally 10^5^ up to more than 10^8^ CFU/ml after 12 h of incubation time (data not shown).

### PRRSV infection weakly impairs *S*. *suis* phagocytosis by porcine BMDCs but not intracellular survival

**Fig 2:**
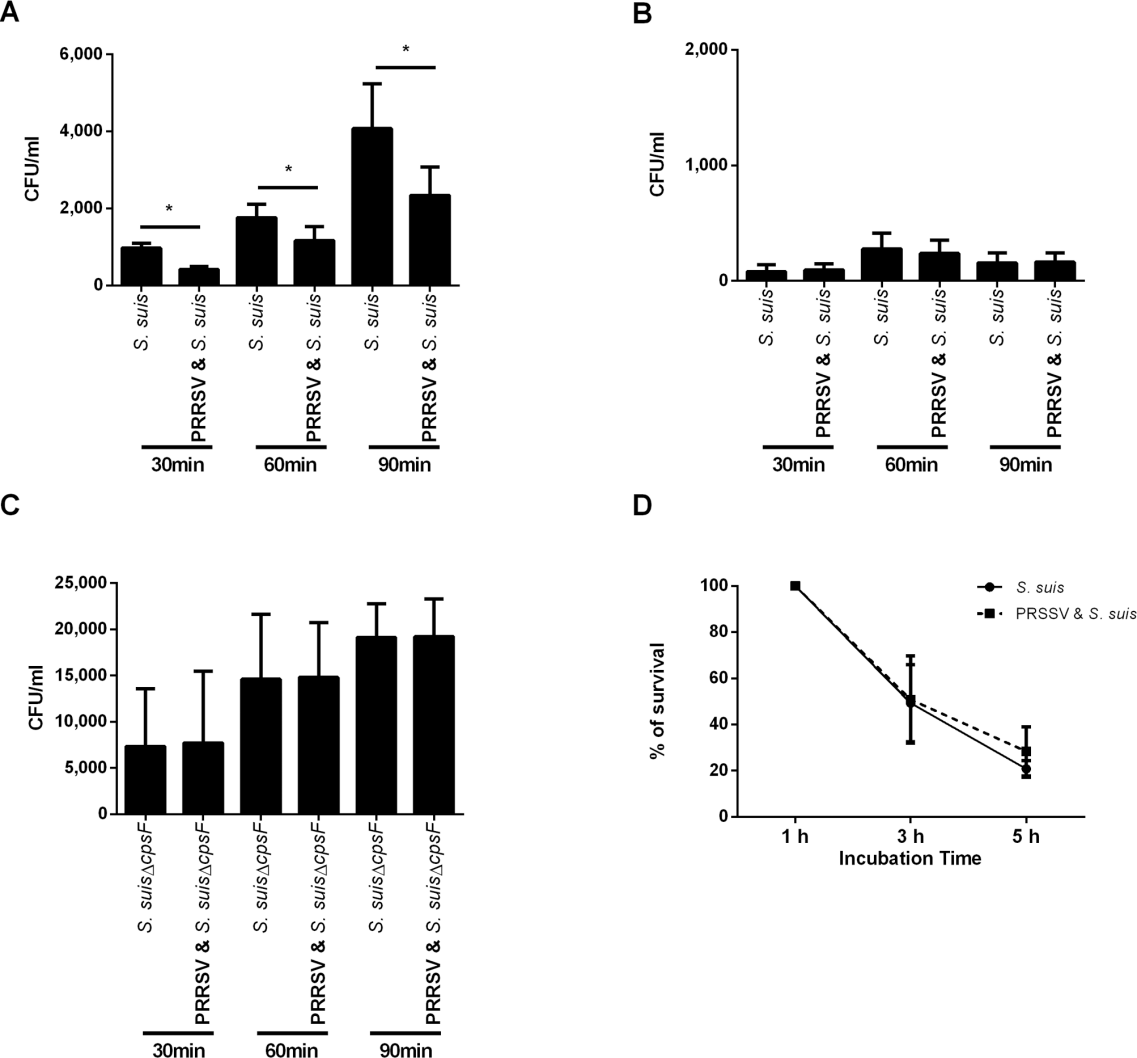
Effect of PRRSV infection on internalization of *S*. *suis* by porcine bone marrow-derived dendritic cells (BMDCs) and monocytes and its intracellular survival. Cells were pre-infected with PRRSV or mock-infected. (A, B) Cells were incubated for 30 min, 60 min or 90 min to assess phagocytosis. (A) Porcine BMDC internalization of wild-type *S*. *suis*. (B) Porcine monocyte internalization of wild-type *S*. *suis*. (C) Internalization of *S*. *suis* non-encapsulated mutant (Δ*cpsf*) by porcine monocytes. (D) *S*. *suis* intracellular cell survival in PRRSV-infected or mock-infected porcine BMDCs. The results show the percentage of viable bacteria, 100% being the number of viable bacteria recovered after 1 h of culture.

In a previous study [11], it was shown that porcine BMDCs could take up *S*. *suis*, even though the capsule conferred a significant resistance to phagocytosis. The number of internalized *S*. *suis* by BMDCs found during this experiment is in agreement with that previously reported [11], with the number of phagocytized bacteria significantly increasing from 30 min to 90 min (Fig 2A). When porcine BMDCs were pre-infected with PRRSV, a significantly lower number of bacteria were internalized after all incubation times (Fig 2A). Monocytes were able to very weakly phagocyte *S*. *suis*, and no difference was seen in the number of internalized bacteria when cells were pre-infected with PRRSV for any of the time points (Fig 2B). Since the number of monocyte-internalized bacteria was too low, we decided to confirm this result by using a non-encapsulated mutant (*S*. *suis* Δ*cps2F*) derived from the *S*. *suis* strain P1/7, which had been previously shown to be more phagocytosed than its corresponding wild-type strain [11]. As expected, a significantly greater number of non-encapsulated *S*. *suis* were internalized by porcine monocytes at all incubation times (*P* < 0.05); however, the number of ingested bacteria was not modified by a PRRSV-pre-infection (Fig 2C).

If phagocytosed by APCs, bacteria have to be killed and processed for antigen presentation to other cells of the immune system. To assess if PRRSV infection had an effect on the intracellular survival of *S*. *suis*, the phagocytosis assay was modified as explained in material and methods. The number of internalized bacteria in monocytes was too low to perform intracellular survival studies (data not shown). In order to compare the intracellular survival in mock-infected and PRRSV-infected cells, the data were normalized and a value of 100% was set for the number of surviving bacteria found after 1 h of internalization (Fig 2D). After 3 h, half of the bacteria were killed and no difference was observed between PRRSV-infected and mock-infected BMDCs (49.3 ± 16.7% and 50.9 ± 19% respectively; *P* > 0.05). Bacterial killing increased until 5 h of culture for both treatments, with around 20% of survival at this last time point. These results suggest that a prior infection with PRRSV would weakly but significantly impair the uptake of bacteria by porcine DCs, but that it does not have any effect on *S*. *suis* killing following phagocytosis.

### General microarray data analysis

**Fig 3:**
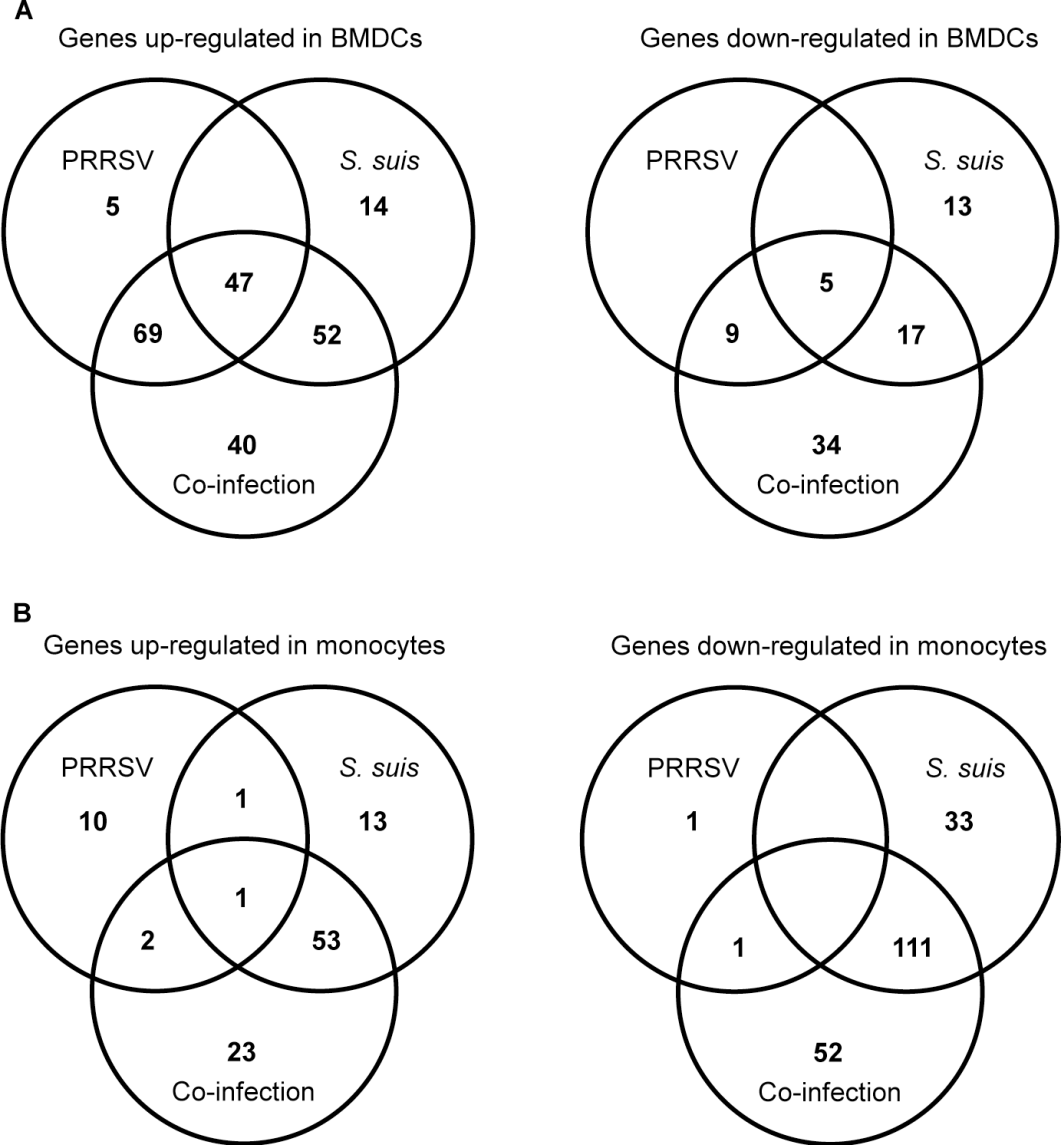
Number of differentially expressed genes in porcine monocytes and bone marrow-derived dendritic cells (BMDCs) following infection with PRRSV, *S*. *suis*, or co-infection with these two pathogens. Venn diagrams representing the number of up-regulated and down-regulated genes in porcine BMDCs (A) and porcine monocytes (B) following 12 h of infection with PRRSV alone, *S*. *suis* alone and co-infection, as determined by the Agilent microarray study. Genes were considered differentially expressed when a fold change greater than 2-fold (up-regulation or down-regulation) and a *P* value ≤ 0.05 was observed compared to the expression in mock-infected corresponding cells.

An incubation time of 12 h after bacterial infection of virus pre-infected cells was chosen based on preliminary data. In fact, earlier incubation times presented low and inconstant results and longer incubation times revealed high cell toxicity (data not shown). Data from infected cells were compared to mock-infected cells. Using an expression threshold ≥ 2 fold with a *P* < 0.05, a total of 113 and 35 genes were up- and down-regulated in BMDCs following *S*. *suis* infection, respectively (Fig 3A). When cells were pre-infected with PRRSV, more than 270 genes were either up- or down-regulated. Among these genes, only a few (40 out of 208) were found to be up-regulated only in the presence of both pathogens, whereas more than half of the down-regulated genes (34 out of 65) were specific to the co-infection (Fig 3A). Differently from BMDCs, monocytes infected by *S*. *suis* alone showed more down-regulated (144) than up-regulated (68) genes (Fig 3B). This was also found in the presence of both pathogens, with 79 up-regulated genes and 164 down-regulated genes (Fig 3B). Most of these genes were also regulated in monocytes infected with *S*. *suis* alone (53 out of 79 for the up-regulated genes and 111 out of 164 for the down-regulated genes), although some up- and down-regulated genes that were specific to PRRSV and *S*. *suis* co-infection (23 and 52 genes, respectively) were also observed (Fig 3B). General response to virus infection was higher in BMDCs than in monocytes (135 and 16 genes modulated, respectively). These results reflect the relatively low response of monocytes, which was probably the consequence of a lower viral infection rate (Fig 1). The complete list of genes is shown in Additional file 1: S1, S2, S3 and S4 Tables.

**Fig 4:**
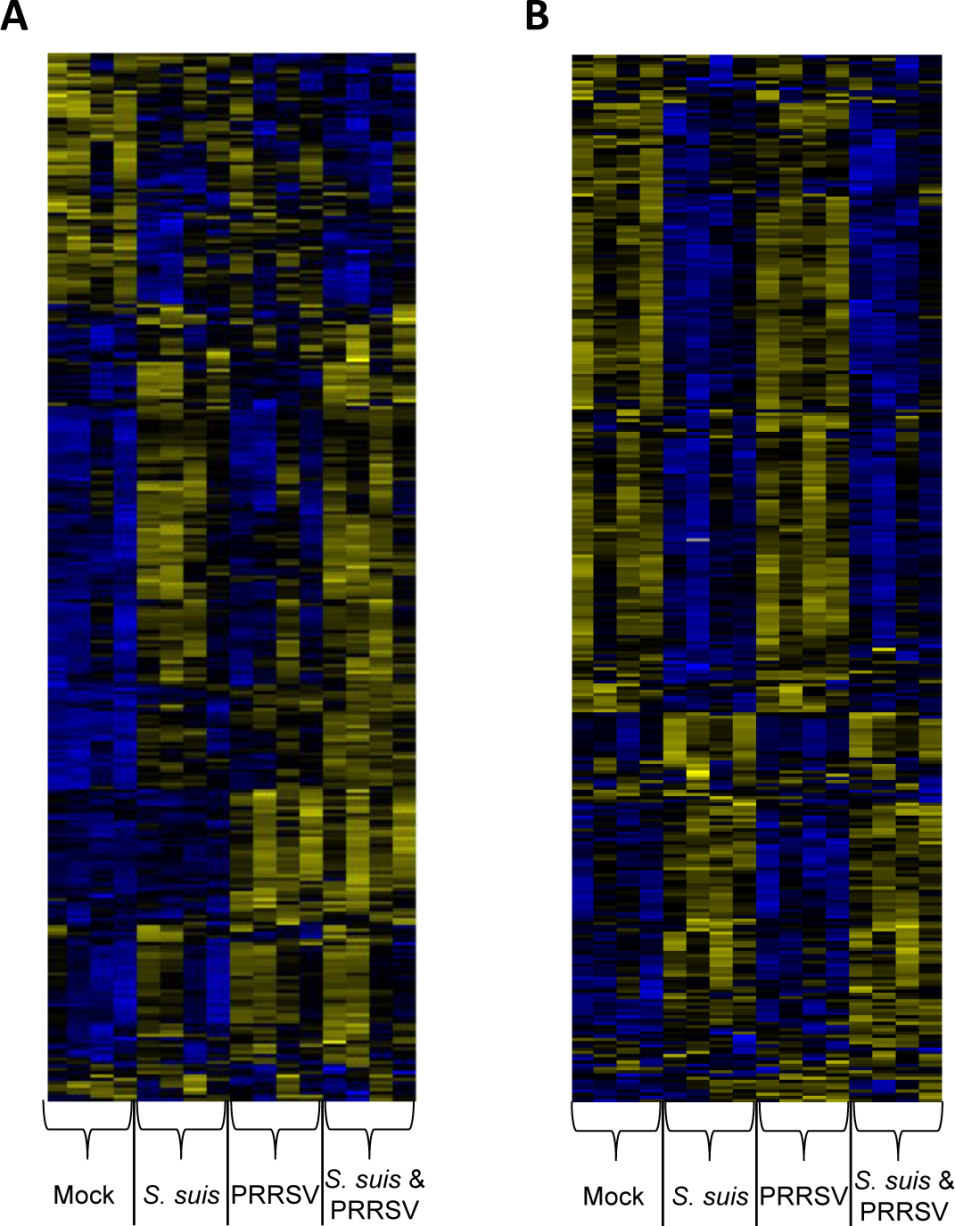
Heat-map of hierarchical clustering analysis of differentially expressed genes. Genes differentially expressed in porcine bone marrow-derived dendritic cells (BMDCs) (A) or monocytes (B) were subjected to hierarchical clustering using the complete linkage method with uncentered correlation as similarity measure. Each row represents a different gene and columns represent individual samples from mock-, *S*. *suis*-, PRRSV- or PRRSV/*S*. *suis*-infected cells from 3 different animals. Bright blue indicates a low expression while bright yellow indicates a high expression.

An unsupervised hierarchical clustering of differentially expressed genes was performed with BMDCs (Fig 4A) and monocytes (Fig 4B). Clustering of the genes displayed different patterns of gene regulation in co-infected BMDCs compared to *S*. *suis*-infected BMDCs. Many clusters of genes regulated by *S*. *suis* or PRRSV alone were also regulated in co-infected cells (Fig 4A). Clustering of the genes in monocytes showed that most genes regulated during co-infection were, in fact, regulated by *S*. *suis*, thus confirming the lower effect of the viral infection on this cell type (Fig 4B).

### Gene Ontology

**Fig 5:**
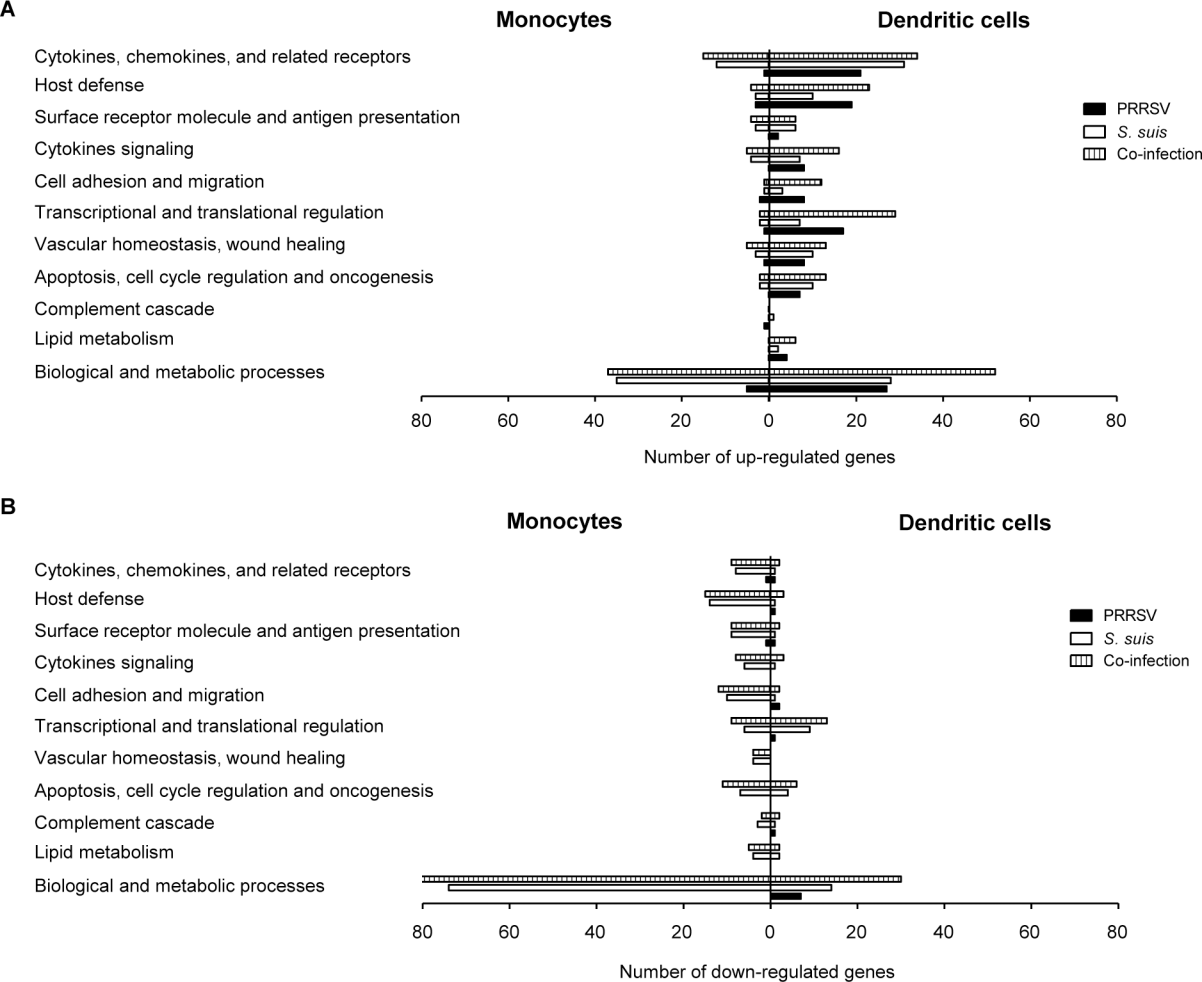
Gene ontology analysis of the differentially expressed genes in porcine bone marrow-derived dendritic cells (BMDCs) and porcine monocytes following PRRSV infection, *S*. *suis* infection and co-infection with the two pathogens. (A) Up-regulated and (B) down-regulated genes in monocytes and BMDCs infected with PRRSV, *S*. *suis* or co-infected with both pathogens.

Genes whose expression was upregulated or downregulated were sorted in different functional groups according to gene ontology as shown in Fig 5. Similar categories of genes were modulated in both cell types, although a higher level of up-regulation could be observed in BMDCs (Fig 5A). In both monocytes and BMDCs, *S*. *suis* infection in the presence or absence of a prior PRRSV infection clearly up-regulated the family of inflammatory-related receptor genes. Host defense associated genes were highly induced by viral infection, with an even greater number of up-regulated genes in *S*. *suis*/PRRSV co-infected BMDCs (Fig 5A). Other categories of up-regulated genes in co-infected BMDCs were those associated to transcriptional and translational regulation and biological and metabolic processes and they will not be further analyzed in this study. On the other hand, down-regulated genes were mostly observed in monocytes activated by *S*. *suis* in the presence or absence of PRRSV (Fig 5B). Down-regulation of genes associated with transcriptional and translational regulations was found to be down-regulated in co-infected or *S*. *suis*-infected BMDCs.

### Cytokine and chemokine gene regulation and inflammatory response

Genes coding for cytokines, chemokines, or molecules involved in cytokine signaling were mostly regulated in BMDCs and, to a lesser extent, in monocytes. Especially for BMDCs, *S*. *suis* infection or co-infection induced the expression of a large panel of genes involved in the recruitment of innate immune cells and the inflammatory response, whereas PRRSV alone or in co-infection induced the expression of type I IFNs and IFN-inducible genes. More specifically, BMDCs infected with *S*. *suis* showed a clear upregulation of CCL3L1, CCL4/MIP-1β and CCL14/HCC-1 (known to attract and/or activate monocytes, macrophages and/or immature DCs), CCL5/RANTES, and CCL8/MCP-2 (chemotactic for monocytes, macrophages, immature DCs, basophils, and activated T cells) as well as CCL20/MIP-3α (known to recruit immature DCs and memory T cells) (S1 Table). In addition, CXC- chemokines such as CXCL2/MIP-2α, CXCL5/AMCF-II and CXCL8/IL-8 (chemotactic for polymorphonuclear cells), were also expressed at higher levels in BMDCs infected with *S*. *suis*. The bacterial infection induced the overexpression of IL-6, TNF-α, IL-1β, IL-15, IL-12p40, and IL-23p19 (the two last being the two subunits of IL-23) (S1 Table).

Following PRRSV/*S*. *suis* co-infection, all of the above mentioned genes were still up-regulated (S1 Table). In fact, an additive effect could be observed for inflammatory mediators such as CCL4, CCL14, CCL20, and IL-15. Interestingly, a clear synergistic effect on IL-6, CCL5 and TNF-α up-regulation could be observed after co-infection (S1 Table). PTGS2 (COX-2), another pro-inflammatory gene involved in the prostaglandin pathway, was also induced in BMDCs (and to a lesser extent in monocytes) infected with *S*. *suis* (S1 and S3 Tables). Since this gene was also up-regulated by PRRSV infection in BMDCs, an additive effect could be observed in co-infected cells (S1 Table).

Type I IFNs are key cytokines playing a pivotal role in the anti-viral response and may also be involved in the response to bacterial infections [44]. Type I IFNs (genes coding for IFN-α and IFN-β) were found to be highly up-regulated by PRRSV and similar levels were observed in co-infected BMDCs. Interestingly, IFN-β expression was also clearly up-regulated by *S*. *suis* (S1 and S2 Tables). IRF-1, a transcription factor responsible for the expression of IFN-inducible genes, was also up-regulated in BMDC stimulated by both pathogens.

*S*. *suis* stimulated monocytes presented up-regulation of very few chemokine/cytokine genes, such as CCL5, CCL20, IL-12p40, and IL-23p19 (S3 Table). PRRSV pre-infection of cells did not influence, in general, the activation of such genes by *S*. *suis*. Conversely, some genes implicated in the inflammatory response were downregulated following stimulation by *S*. *suis* or both pathogens, such as those coding for the chemokine receptors CCR3 (receptor for CCL5, -11, -13, and -28) and CXCR2 (receptor for CXCL1, -2, -3, -5, -7, and -8) (S4 Table).

### Genes associated with host defense

Most of modified genes associated with host defense were up-regulated in BMDCs by PRRSV infection only and *S*. *suis* appeared to have little effect on the expression of such genes. Amongst them, IRG-6 and GBP-1 code for molecules that adhere to pathogen-containing vacuoles to inhibit the intracellular growth of these pathogens [43]. The upregulation of genes from the IFIT family, which are genes coding for anti-viral proteins interfering with virus replication [44], as well as three other anti-viral genes: MX1, MX2 and OAS, was observed. PRRSV and *S*. *suis* co-infection also induced the expression of DDX58 and IFIH1, two members of the RIG-I-like receptors (RLRs) family, coding for RIG-I and MDA5, which are cytoplasmic sensors of viral infections. The triggering of these receptors recognizing PAMPs leads to the production of type I IFNs and pro-inflammatory cytokines. Although *S*. *suis* was not able to up-regulate NOD2, it induced (in presence or absence of PRRSV infection) genes responsible for NLRP3, a receptor which recognizes damage-associated molecular patterns.

### Validation of microarray data by real-time qPCR

The expression levels of selected genes found to be differentially expressed by Agilent microarray assay were confirmed using real-time qPCR analysis in order to validate the microarray assay results. The expression of 10 genes upregulated in BMDCs were studied (Table 2). Three of these genes (IL-12p40, IL-23p19 and CCL20) were also tested in monocytes. The majority of the genes tested were modified in similar proportions to those observed in the microarray study (Table 2). In fact, a significant correlation of the RT-qPCR data to the microarray data was observed with a Pearson R value of 0.9832 (R^2^ = 0.9667, P *<* 0.0001). Small differences sometimes observed could be due to different sensitivity and design of the probes or the primers used in the microarray and the real-time qPCR experiments.

**Table 2:** Validation of microarray gene expression using real-time qPCR in porcine bone-marrow derived dendritic cells (BMDCs) and monocytes infected with PRRSV, *S*. *suis*, or co-infected with both PRRSV and *S*. *suis*.

Gene	Quantification method	Fold increase in expression		
		PRRSV	*S*. *suis*	PRRSV & *S*. *suis*
**BMDCs**				
**Il-6**	Microarray	3.8	35.9	51.8
	qPCR	6.6 ± 2.2	117.8 ± 63.2	150.5 ± 67.2
**CXCL8**	Microarray	1.5	6.6	7.0
	qPCR	1.9 ± 0.4	11.6 ± 4.0	10.1 ± 2.4
**IL-12p40**	Microarray	1.9	3.3	5.7
	qPCR	2.7 ± 0.4	2.9 ± 0.7	7.1 ± 2.4
**IL-23p19**	Microarray	1.8	26.4	29.2
	qPCR	2.6 ± 0.4	3.9 ± 1.5	4.2 ± 0.6
**IL-15**	Microarray	3.1	2.1	4.9
	qPCR	4.7 ± 1.6	2.4 ± 0.6	8.3 ± 3.6
**TNF**	Microarray	8.6	26.5	45.1
	qPCR	10.6 ± 3.1	19.7 ± 5.6	42.5 ± 11.8
**IFN-β**	Microarray	300.9	10.9	308.7
	qPCR	853.1 ± 286.1	17.1 ± 6.3	696.8 ± 240.9
**CCL20**	Microarray	6.5	37.3	47.5
	qPCR	8.2 ± 1.8	40.0 ± 16.2	57.4 ± 18.9
**COX-2**	Microarray	6.5	5.3	11.0
	qPCR	10.2 ± 3.7	5.7 ± 1.1	14.0 ± 3.2
**NOD-2**	Microarray	2.4	1.9	3.1
	qPCR	2.9 ± 0.5	1.8 ± 0.3	3.1 ± 0.5
**Monocytes**				
**Il-12p40**	Microarray	-1.3	19.4	25.8
	qPCR	1.0 ± 0.3	7.4 ± 2.2	7.5 ± 2.2
**IL-23p19**	Microarray	-1.3	10.1	16.5
	qPCR	-1.1 ± 0.4	6.6 ± 1.9	12.9 ± 2.3
**CCL20**	Microarray	1.4	5.0	9.6
	qPCR	1.6 ± 0.7	4.6 ± 1.0	11.7 ± 3.2

## Discussion

Co-infection with PRRSV and *S*. *suis* generally induces exacerbated disease leading to an increased morbidity and mortality [24]. Despite the important economic impact of this co-infection on the porcine industry, few studies have examined the pathogenesis of the concurrent infections and few data on the immune mechanisms that may lead to the greater susceptibility of co-infected animals are available. In the present study, we evaluated the effect of a PRRSV infection on the response to a subsequent *S*. *suis* co-infection with two types of cells: BMDCs and monocytes. We mainly focused the analysis in our study on the inflammatory genes that may be affected during a co-infection. In the bloodstream, PRRSV comes in contact with both cell populations that display different susceptibility to PRRSV infection. While porcine DCs have been reported to be very susceptible to PRRSV infection [44], porcine monocytes may be more refractory to it [45]. Similar results were obtained in the present study, since BMDC were clearly more permissive to virus replication than monocytes.

Once *S*. *suis* reaches deep tissues and bloodstream, it is subjected to the action of phagocytic cells of the immune system. However, in the absence of specific antibodies, it is relatively able to resist phagocytosis and persists in the blood with important inflammatory consequences [9]. *S*. *suis* also interacts with DCs and monocytic cells [10,11], and a previous infection with PRRSV may have an important influence on cell responses. It has been described that porcine DCs are able, to a certain extent, to internalize well-encapsulated *S*. *suis*, despite the anti-phagocytic role of the CPS [11]. Results obtained in this study confirmed such observations, where phagocytosis seems to be time-dependent. However, a pre-infection with PRRSV significantly reduced (around 50%) the internalization of encapsulated *S*. *suis* by BMDCs, though it did not affect the killing rate of intracellular bacteria. It is not clear if these differences observed *in vitro* may have a real influence on the level of antigen processing *in vivo*. Previous studies showed a decreased phagocytic activity of beads and dextran by PRRSV-infected porcine pulmonary alveolar macrophages and monocyte-derived DCs [45,46]. Interestingly, this is the first study showing the effect of PRRSV on the phagocytic properties of DCs using live bacterial pathogens. Very few studies reported the effect of PRRSV on the phagocytic properties of PAMs against live extracellular bacteria. More precisely, a pre-infection of pulmonary alveolar macrophages with PRRSV induced either a decreased activity or had no impact on their ability to ingest and kill *Haemophilus parasuis*, depending on the study [25,26]. To date, no study had been carried out with PRRSV/*S*. *suis* co-infected cells. On the other hand, *S*. *suis* was almost not phagocytosed by monocytes and a pre-infection with PRRSV did not change such observations. Although no differences could be observed between either virus-infected and control cells when a non-encapsulated mutant was used, it remains unclear if herein obtained results are simply related to the low PRRSV infectivity of monocytes. It should be noted that results from the present study are *in vitro*, whereas *in vivo*, other factors may also influence the phagocytosis of bacteria by professional phagocytic cells.

A handful of studies investigated the modification of gene expression following *in vitro* infection of monocytes, pulmonary alveolar macrophages or DCs by PRRSV, some of these using whole transcriptional analyses [47,48]. Very few studies have analyzed the complete gene expression profiles of monocytes or alveolar macrophages activated by *S*. *suis* [49,50]. Yet, none has determined the activation profile of *S*. *suis*-infected DCs. In addition, the transcriptional immune response of porcine cells after a co-infection with PRRSV and *S*. *suis* had not been previously addressed. Gene ontology showed that the main groups of regulated genes were pro-inflammatory mediators and related receptors, as well as host defense genes. Regulated genes of these groups were the main focus of this study. PRRSV induced differential expression of a higher number of genes in BMDCs than in monocytes, as a possible consequence of a lower virus infectivity of the latter cell type. As previously mentioned, it has been reported that monocytes may not be optimally infected by some strains of PRRSV [45].

The innate immune response is the first line of defense against infection [51]. PRRSV infection of cells clearly up-regulated IFN-β and, to a lesser extent, IFN-α, confirming previous published data [52,53]. Interestingly, *S*. *suis* also up-regulated IFN-β and, to a lesser extent, IFN-α. Although the up-regulation of type I IFN genes has previously been reported *in vitro* with swine epithelial cells [54] and *in vivo* in a mouse model of infection [16], this is the first report of a clear up-regulation of such genes by porcine DCs infected with *S*. *suis*. Type I IFNs are now known to play important roles in infections caused by extracellular bacteria, including streptococci [55]. Although the co-infection did not potentiate the expression of type I IFNs compared to cells infected with PRRSV alone, the elevated production of these cytokines by PRRSV could have an incidence on the bacterial infection. IFN-β has been shown to potentiate the septic shock syndrome or to induce the inhibition of the myeloid cell response to IFN-γ, an important cytokine for bacterial clearance [56,57]. However, this hypothesis remains to be confirmed.

One of the hallmarks of the disease caused by *S*. *suis* is an excessive inflammatory host reaction during both systemic (septic shock) and central nervous system (meningitis) infections. Previous studies showed that highly virulent *S*. *suis* serotype 2 strains induced high levels of pro-inflammatory cytokines, which resulted in a cytokine storm that induced higher susceptibility to the infection [16,58]. *In vitro* results obtained in the present study confirmed that BMDCs infected by *S*. *suis* induce the up-regulation of potent inflammatory mediators. When cells were pre-infected with PRRSV, a clear additional or synergistic effect on the expression of chemokines and pro-inflammatory cytokines could be observed when compared to cells infected by a single pathogen. We recently reported that *in vitro* co-infection of swine epithelial cells with *S*. *suis* and swine influenza virus showed an important synergy between the two pathogens regarding the up-regulation of genes coding for inflammatory mediators [54]. A recent study carried out *in vivo* confirmed such observations, also showing a clear increase in clinical signs observed in co-infected animals [59]. More specifically, PRRSV/*S*. *suis* co-infection potentiates the up-regulation of IL-6, CCL5 and TNF-α. Interestingly, it has already been reported that *S*. *suis* is able to induce the production of CCL5 *in vivo* and *in vitro* [15,60], and this expression was also synergistically increased when cells were pre-infected with swine influenza virus [54]. Both TNF-α and IL-6 are known to be key cytokines in the inflammatory response and their over-expression in PRRSV and *S*. *suis* co-infected cells could suggest an increased pro-inflammatory response detrimental for co-infected animals. Moreover, the increased expression of the chemokine CCL5 could participate in a positive feedback loop of the inflammatory response by enhancing the recruitment of macrophages, immature DCs or basophils.

Prostanoids (prostaglandins and thromboxane A2) appear to be important regulators of both the onset and resolution of the inflammatory response. These molecules derive from arachidonic acid, an unsaturated fatty acid that is released from the plasma membrane by phospholipases in response to several activation stimuli, including pro-inflammatory mediators [61]. It is subsequently metabolized by cyclooxygenases (COXs) and lipoxygenases to generate, among other products, prostaglandins (PG). The inducible enzyme COX-2 (PTGS2 gene), which catalyzes the first step in the biosynthesis of PGs from arachidonic acid, is known to contribute to the acute phase of inflammation, especially by catalysing PGE_2_ production. Recent results showed that *S*. *suis* infection results in a shift toward an induction of pro-inflammatory lipid mediators and that treatment with fenretinide is able to reduce the levels of arachidonic acid and hence, reduce the excessive pro-inflammatory response *in vivo* [16]. In addition, *S*. *suis* is able to induce the secretion of PGE_2_ by human macrophages [62]. In the present study, both PRRSV and *S*. *suis* up-regulated the expression of COX-2 and the effect was clearly additive when the two pathogens acted together. A synergism concerning this mediator was recently observed in *S*. *suis*/swine influenza virus co-infected epithelial cells [54]. It may be hypothesized that lipid mediators play an important role in inflammation during *S*. *suis* infection and this role would be amplified during co-infections with important porcine pathogenic viruses.

Co-infections of PRRSV/*S*. *suis* leading to an exacerbated inflammation would confirm previous results obtained in PRRSV co-infections. For example, cells co-infected with PRRSV and porcine circovirus produced more TNF than cells infected with either pathogen [63,64]. An exacerbated inflammatory response was also described in pigs co-infected with PRRSV and *Mycoplasma hyopneumoniae* [65]. A clear synergism of the inflammatory response was also observed between PRRSV and LPS [66–68]. However, results obtained in the present study should still be confirmed *in vivo*.

In conclusion, this study suggests that PRRSV infection can modulate the innate immune system by partially acting on the phagocytosis of *S*. *suis*, but mainly by modulating the development of an exacerbate inflammatory response. DCs, more susceptible to virus infection, are important players in such response. The results of the present study are the first step in understanding the immunological mechanisms explaining the higher susceptibility of PRRSV-infected animals to *S*. *suis*. *In vivo* studies, presently underway, will help to better understand the mechanisms involved during co-infections with these two important swine pathogens.

## Supporting Information

S1 Table Genes upregulated greater than two-fold in porcine BMDCs after infection by PRRSV, *S*. *suis*, or co-infected with both pathogens for 12 h, compared to mock-infected cells. (DOCX)

S2 Table Genes downregulated greater than two-fold in porcine BMDCs after infection by PRRSV, *S*. *suis*, or co-infected with both pathogens for 12 h, compared to mock-infected cells. (DOCX)

S3 Table Genes upregulated greater than two-fold in porcine monocytes after infection by PRRSV, *S*. *suis*, or co-infected with both pathogens for 12 h, compared to mock-infected cells. (DOCX)

S4 Table Genes downregulated greater than two-fold in porcine monocytes infected with PRRSV, *S*. *suis*, or co-infected with both pathogens for 12 h, compared to mock-infected cells. (DOCX)

## References

[1] Lunney JK, Benfield DA, Rowland RR (2010) Porcine reproductive and respiratory syndrome virus: an update on an emerging and re-emerging viral disease of swine. Virus Res 154: 1–6. 10.1016/j.virusres.2010.10.009 20951175 PMC7172856

[2] Kimman TG, Cornelissen LA, Moormann RJ, Rebel JM, Stockhofe-Zurwieden N (2009) Challenges for porcine reproductive and respiratory syndrome virus (PRRSV) vaccinology. Vaccine 27: 3704–3718. 10.1016/j.vaccine.2009.04.022 19464553

[3] Neumann EJ, Kliebenstein JB, Johnson CD, Mabry JW, Bush EJ, Seitzinger AH, et al (2005) Assessment of the economic impact of porcine reproductive and respiratory syndrome on swine production in the United States. J Am Vet Med Assoc 227: 385–392. 16121604 10.2460/javma.2005.227.385

[4] Gomez-Laguna J, Salguero FJ, Pallares FJ, Carrasco L (2013) Immunopathogenesis of porcine reproductive and respiratory syndrome in the respiratory tract of pigs. Vet J 195: 148–155. 10.1016/j.tvjl.2012.11.012 23265866 PMC7128372

[5] Gottschalk M, Xu J, Calzas C, Segura M (2010) Streptococcus suis: a new emerging or an old neglected zoonotic pathogen? Future Microbiol 5: 371–391. 10.2217/fmb.10.2 20210549

[6] MacInnes JI, Gottschalk M, Lone AG, Metcalf DS, Ojha S, Rosendal T, et al (2008) Prevalence of Actinobacillus pleuropneumoniae, Actinobacillus suis, Haemophilus parasuis, Pasteurella multocida, and Streptococcus suis in representative Ontario swine herds. Can J Vet Res 72: 242–248. 18505187 PMC2327245

[7] Goyette-Desjardins G, Auger JP, Xu J, Segura M, Gottschalk M (2014) Streptococcus suis, an important pig pathogen and emerging zoonotic agent-an update on the worldwide distribution based on serotyping and sequence typing. Emerg Microbes Infect 3: e45 10.1038/emi.2014.45 26038745 PMC4078792

[8] Madsen LW, Bak H, Nielsen B, Jensen HE, Aalbaek B, Riising HJ (2002) Bacterial colonization and invasion in pigs experimentally exposed to Streptococcus suis serotype 2 in aerosol. Journal of veterinary medicine B, Infectious diseases and veterinary public health 49: 211–215. 12121040 10.1046/j.1439-0450.2002.00491.x

[9] Fittipaldi N, Segura M, Grenier D, Gottschalk M (2012) Virulence factors involved in the pathogenesis of the infection caused by the swine pathogen and zoonotic agent Streptococcus suis. Future Microbiol 7: 259–279. 10.2217/fmb.11.149 22324994

[10] Gottschalk M, Segura M (2000) The pathogenesis of the meningitis caused by Streptococcus suis: the unresolved questions. Vet Microbiol 76: 259–272. 10973700 10.1016/s0378-1135(00)00250-9

[11] Lecours MP, Segura M, Lachance C, Mussa T, Surprenant C, Montoya M, et al (2011) Characterization of porcine dendritic cell response to Streptococcus suis. Vet Res 42: 72 10.1186/1297-9716-42-72 21635729 PMC3127767

[12] Chabot-Roy G, Willson P, Segura M, Lacouture S, Gottschalk M (2006) Phagocytosis and killing of Streptococcus suis by porcine neutrophils. Microb Pathog 41: 21–32. 16714092 10.1016/j.micpath.2006.04.001

[13] Smith HE, Damman M, van der Velde J, Wagenaar F, Wisselink HJ, Stockhofe-Zurwieden N, et al (1999) Identification and characterization of the cps locus of Streptococcus suis serotype 2: the capsule protects against phagocytosis and is an important virulence factor. Infect Immun 67: 1750–1756. 10085014 10.1128/iai.67.4.1750-1756.1999 PMC96524

[14] Williams AE (1990) Relationship between intracellular survival in macrophages and pathogenicity of Streptococcus suis type 2 isolates. Microb Pathog 8: 189–196. 2199755 10.1016/0882-4010(90)90046-s

[15] Dominguez-Punaro MC, Segura M, Plante MM, Lacouture S, Rivest S, Gottschalk M (2007) Streptococcus suis serotype 2, an important swine and human pathogen, induces strong systemic and cerebral inflammatory responses in a mouse model of infection. J Immunol 179: 1842–1854. 17641051 10.4049/jimmunol.179.3.1842

[16] Lachance C, Gottschalk M, Gerber PP, Lemire P, Xu J, Segura M (2013) Exacerbated type II interferon response drives hypervirulence and toxic shock by an emergent epidemic strain of Streptococcus suis. Infect Immun 81: 1928–1939. 10.1128/IAI.01317-12 23509145 PMC3676015

[17] Graveline R, Segura M, Radzioch D, Gottschalk M (2007) TLR2-dependent recognition of Streptococcus suis is modulated by the presence of capsular polysaccharide which modifies macrophage responsiveness. Int Immunol 19: 375–389. 17307800 10.1093/intimm/dxm003

[18] Segura M, Vadeboncoeur N, Gottschalk M (2002) CD14-dependent and -independent cytokine and chemokine production by human THP-1 monocytes stimulated by Streptococcus suis capsular type 2. Clin Exp Immunol 127: 243–254. 11876746 10.1046/j.1365-2249.2002.01768.x PMC1906344

[19] Opriessnig T, Halbur PG (2012) Concurrent infections are important for expression of porcine circovirus associated disease. Virus Res 164: 20–32. 10.1016/j.virusres.2011.09.014 21959087 PMC7114432

[20] Van Reeth K, Nauwynck H, Pensaert M (1996) Dual infections of feeder pigs with porcine reproductive and respiratory syndrome virus followed by porcine respiratory coronavirus or swine influenza virus: a clinical and virological study. Vet Microbiol 48: 325–335. 9054128 10.1016/0378-1135(95)00145-X PMC7117459

[21] Yu J, Wu J, Zhang Y, Guo L, Cong X, Du Y, et al (2012) Concurrent highly pathogenic porcine reproductive and respiratory syndrome virus infection accelerates Haemophilus parasuis infection in conventional pigs. Vet Microbiol 158: 316–321. 10.1016/j.vetmic.2012.03.001 22460022

[22] Brockmeier SL, Palmer MV, Bolin SR (2000) Effects of intranasal inoculation of porcine reproductive and respiratory syndrome virus, Bordetella bronchiseptica, or a combination of both organisms in pigs. Am J Vet Res 61: 892–899. 10951978 10.2460/ajvr.2000.61.892

[23] Thacker EL, Halbur PG, Ross RF, Thanawongnuwech R, Thacker BJ (1999) Mycoplasma hyopneumoniae potentiation of porcine reproductive and respiratory syndrome virus-induced pneumonia. J Clin Microbiol 37: 620–627. 9986823 10.1128/jcm.37.3.620-627.1999 PMC84495

[24] Thanawongnuwech R, Brown GB, Halbur PG, Roth JA, Royer RL, Thacker BJ (2000) Pathogenesis of porcine reproductive and respiratory syndrome virus-induced increase in susceptibility to Streptococcus suis infection. Vet Pathol 37: 143–152. 10714643 10.1354/vp.37-2-143

[25] Solano GI, Bautista E, Molitor TW, Segales J, Pijoan C (1998) Effect of porcine reproductive and respiratory syndrome virus infection on the clearance of Haemophilus parasuis by porcine alveolar macrophages. Can J Vet Res 62: 251–256. 9798089 PMC1189490

[26] Segales J, Domingo M, Balasch M, Solano GI, Pijoan C (1998) Ultrastructural study of porcine alveolar macrophages infected in vitro with porcine reproductive and respiratory syndrome (PRRS) virus, with and without Haemophilus parasuis. J Comp Pathol 118: 231–243. 9595354 10.1016/s0021-9975(05)80129-x

[27] Galina L, Pijoan C, Sitjar M, Christianson WT, Rossow K, Collins JE (1994) Interaction between Streptococcus suis serotype 2 and porcine reproductive and respiratory syndrome virus in specific pathogen-free piglets. Vet Rec 134: 60–64. 8135015 10.1136/vr.134.3.60

[28] Xu M, Wang S, Li L, Lei L, Liu Y, Shi W, et al (2010) Secondary infection with Streptococcus suis serotype 7 increases the virulence of highly pathogenic porcine reproductive and respiratory syndrome virus in pigs. Virol J 7: 184 10.1186/1743-422X-7-184 20696031 PMC2927530

[29] Halbur P, Thanawongnuwech R, Brown G, Kinyon J, Roth J, Thacker E, et al (2000) Efficacy of antimicrobial treatments and vaccination regimens for control of porcine reproductive and respiratory syndrome virus and Streptococcus suis coinfection of nursery pigs. J Clin Microbiol 38: 1156–1160. 10699012 10.1128/jcm.38.3.1156-1160.2000 PMC86362

[30] Feng W, Laster SM, Tompkins M, Brown T, Xu JS, Altier C, et al (2001) In utero infection by porcine reproductive and respiratory syndrome virus is sufficient to increase susceptibility of piglets to challenge by Streptococcus suis type II. J Virol 75: 4889–4895. 11312360 10.1128/JVI.75.10.4889-4895.2001 PMC114243

[31] Banchereau J, Briere F, Caux C, Davoust J, Lebecque S, Liu YJ, et al (2000) Immunobiology of dendritic cells. Annu Rev Immunol 18: 767–811. 10837075 10.1146/annurev.immunol.18.1.767

[32] Geissmann F, Manz MG, Jung S, Sieweke MH, Merad M, Ley K (2010) Development of monocytes, macrophages, and dendritic cells. Science 327: 656–661. 10.1126/science.1178331 20133564 PMC2887389

[33] Holden MT, Hauser H, Sanders M, Ngo TH, Cherevach I, Cronin A, et al (2009) Rapid evolution of virulence and drug resistance in the emerging zoonotic pathogen Streptococcus suis. PLoS One 4: e6072 10.1371/journal.pone.0006072 19603075 PMC2705793

[34] Lecours MP, Fittipaldi N, Takamatsu D, Okura M, Segura M, Goyette-Desjardins G, et al (2012) Sialylation of Streptococcus suis serotype 2 is essential for capsule expression but is not responsible for the main capsular epitope. Microbes Infect 14: 941–950. 10.1016/j.micinf.2012.03.008 22521569

[35] Dea S, Gagnon CA, Mardassi H, Milane G (1996) Antigenic variability among North American and European strains of porcine reproductive and respiratory syndrome virus as defined by monoclonal antibodies to the matrix protein. J Clin Microbiol 34: 1488–1493. 8735103 10.1128/jcm.34.6.1488-1493.1996 PMC229047

[36] Meier WA, Galeota J, Osorio FA, Husmann RJ, Schnitzlein WM, Zuckermann FA (2003) Gradual development of the interferon-gamma response of swine to porcine reproductive and respiratory syndrome virus infection or vaccination. Virology 309: 18–31. 12726723 10.1016/s0042-6822(03)00009-6

[37] Auray G, Facci MR, van Kessel J, Buchanan R, Babiuk LA, Gerdts V (2013) Porcine neonatal blood dendritic cells, but not monocytes, are more responsive to TLRs stimulation than their adult counterparts. PLoS One 8: e59629 10.1371/journal.pone.0059629 23667422 PMC3648567

[38] Gagnon CA, del Castillo JR, Music N, Fontaine G, Harel J, Tremblay D (2008) Development and use of a multiplex real-time quantitative polymerase chain reaction assay for detection and differentiation of Porcine circovirus-2 genotypes 2a and 2b in an epidemiological survey. J Vet Diagn Invest 20: 545–558. 18776085 10.1177/104063870802000503

[39] Bustin SA, Benes V, Garson JA, Hellemans J, Huggett J, Kubista M, et al (2009) The MIQE guidelines: minimum information for publication of quantitative real-time PCR experiments. Clin Chem 55: 611–622. 10.1373/clinchem.2008.112797 19246619

[40] Schmittgen TD, Livak KJ (2008) Analyzing real-time PCR data by the comparative C(T) method. Nat Protoc 3: 1101–1108. 18546601 10.1038/nprot.2008.73

[41] Tohno M, Ueda W, Azuma Y, Shimazu T, Katoh S, Wang JM, et al (2008) Molecular cloning and functional characterization of porcine nucleotide-binding oligomerization domain-2 (NOD2). Mol Immunol 45: 194–203. 17559936 10.1016/j.molimm.2007.04.019

[42] de Hoon MJ, Imoto S, Nolan J, Miyano S (2004) Open source clustering software. Bioinformatics 20: 1453–1454. 14871861 10.1093/bioinformatics/bth078

[43] Saldanha AJ (2004) Java Treeview—extensible visualization of microarray data. Bioinformatics 20: 3246–3248. 15180930 10.1093/bioinformatics/bth349

[44] McNab F, Mayer-Barber K, Sher A, Wack A, O'Garra A (2015) Type I interferons in infectious disease. Nat Rev Immunol 15: 87–103. 10.1038/nri3787 25614319 PMC7162685

[45] Wang X, Eaton M, Mayer M, Li H, He D, Nelson E, et al (2007) Porcine reproductive and respiratory syndrome virus productively infects monocyte-derived dendritic cells and compromises their antigen-presenting ability. Arch Virol 152: 289–303. 17031757 10.1007/s00705-006-0857-1

[46] De Baere MI, Van Gorp H, Delputte PL, Nauwynck HJ (2012) Interaction of the European genotype porcine reproductive and respiratory syndrome virus (PRRSV) with sialoadhesin (CD169/Siglec-1) inhibits alveolar macrophage phagocytosis. Vet Res 43: 47 10.1186/1297-9716-43-47 22630829 PMC3403922

[47] Li B, Du L, Xu X, Sun B, Yu Z, Feng Z, et al (2015) Transcription analysis on response of porcine alveolar macrophages to co-infection of the highly pathogenic porcine reproductive and respiratory syndrome virus and Mycoplasma hyopneumoniae. Virus Res 196: 60–69. 10.1016/j.virusres.2014.11.006 25445346

[48] Zhou P, Zhai S, Zhou X, Lin P, Jiang T, Hu X, et al (2011) Molecular characterization of transcriptome-wide interactions between highly pathogenic porcine reproductive and respiratory syndrome virus and porcine alveolar macrophages in vivo. Int J Biol Sci 7: 947–959. 21850204 10.7150/ijbs.7.947 PMC3157269

[49] de Greeff A, Benga L, Wichgers Schreur PJ, Valentin-Weigand P, Rebel JM, Smith HE (2010) Involvement of NF-kappaB and MAP-kinases in the transcriptional response of alveolar macrophages to Streptococcus suis. Vet Microbiol 141: 59–67. 10.1016/j.vetmic.2009.07.031 19709818

[50] Liu M, Tan C, Fang L, Xiao S, Chen H (2011) Microarray analyses of THP-1 cells infected with Streptococcus suis serotype 2. Vet Microbiol 150: 126–131. 10.1016/j.vetmic.2010.12.014 21255946

[51] Levy DE, Marie IJ, Durbin JE (2011) Induction and function of type I and III interferon in response to viral infection. Curr Opin Virol 1: 476–486. 10.1016/j.coviro.2011.11.001 22323926 PMC3272644

[52] Zhang H, Guo X, Nelson E, Christopher-Hennings J, Wang X (2012) Porcine reproductive and respiratory syndrome virus activates the transcription of interferon alpha/beta (IFN-alpha/beta) in monocyte-derived dendritic cells (Mo-DC). Vet Microbiol 159: 494–498. 10.1016/j.vetmic.2012.04.025 22592217 PMC7127654

[53] Lee SM, Schommer SK, Kleiboeker SB (2004) Porcine reproductive and respiratory syndrome virus field isolates differ in in vitro interferon phenotypes. Vet Immunol Immunopathol 102: 217–231. 15507307 10.1016/j.vetimm.2004.09.009 PMC7112598

[54] Dang Y, Lachance C, Wang Y, Gagnon CA, Savard C, Segura M, et al (2014) Transcriptional approach to study porcine tracheal epithelial cells individually or dually infected with swine influenza virus and Streptococcus suis. BMC Vet Res 10: 86 10.1186/1746-6148-10-86 24708855 PMC4022123

[55] Mancuso G, Midiri A, Biondo C, Beninati C, Zummo S, Galbo R, et al (2007) Type I IFN signaling is crucial for host resistance against different species of pathogenic bacteria. J Immunol 178: 3126–3133. 17312160 10.4049/jimmunol.178.5.3126

[56] Karaghiosoff M, Steinborn R, Kovarik P, Kriegshauser G, Baccarini M, Donabauer B, et al (2003) Central role for type I interferons and Tyk2 in lipopolysaccharide-induced endotoxin shock. Nat Immunol 4: 471–477. 12679810 10.1038/ni910

[57] Kearney SJ, Delgado C, Eshleman EM, Hill KK, O'Connor BP, Lenz LL (2013) Type I IFNs Downregulate Myeloid Cell IFN-gamma Receptor by Inducing Recruitment of an Early Growth Response 3/NGFI-A Binding Protein 1 Complex That Silences ifngr1 Transcription. J Immunol 191: 3384–3392. 10.4049/jimmunol.1203510 23935197 PMC3777655

[58] Dominguez-Punaro MC, Segura M, Radzioch D, Rivest S, Gottschalk M (2008) Comparison of the susceptibilities of C57BL/6 and A/J mouse strains to Streptococcus suis serotype 2 infection. Infect Immun 76: 3901–3910. 10.1128/IAI.00350-08 18573893 PMC2519407

[59] Lin X, Huang C, Shi J, Wang R, Sun X, Liu X, et al (2015) Investigation of Pathogenesis of H1N1 Influenza Virus and Swine Streptococcus suis Serotype 2 Co-Infection in Pigs by Microarray Analysis. PLoS One 10: e0124086 10.1371/journal.pone.0124086 25906258 PMC4407888

[60] Bonifait L, Grenier D (2011) The SspA subtilisin-like protease of Streptococcus suis triggers a pro-inflammatory response in macrophages through a non-proteolytic mechanism. BMC Microbiol 11: 47 10.1186/1471-2180-11-47 21362190 PMC3058005

[61] Ricciotti E, FitzGerald GA (2011) Prostaglandins and inflammation. Arterioscler Thromb Vasc Biol 31: 986–1000. 10.1161/ATVBAHA.110.207449 21508345 PMC3081099

[62] Jobin MC, Gottschalk M, Grenier D (2006) Upregulation of prostaglandin E2 and matrix metalloproteinase 9 production by human macrophage-like cells: synergistic effect of capsular material and cell wall from Streptococcus suis. Microb Pathog 40: 29–34. 16324819 10.1016/j.micpath.2005.10.003

[63] Tsai YC, Chang HW, Jeng CR, Lin TL, Lin CM, Wan CH, et al (2012) The effect of infection order of porcine circovirus type 2 and porcine reproductive and respiratory syndrome virus on dually infected swine alveolar macrophages. BMC Vet Res 8: 174 10.1186/1746-6148-8-174 23009687 PMC3528418

[64] Shi KC, Guo X, Ge XN, Liu Q, Yang HC (2010) Cytokine mRNA expression profiles in peripheral blood mononuclear cells from piglets experimentally co-infected with porcine reproductive and respiratory syndrome virus and porcine circovirus type 2. Vet Microbiol 140: 155–160. 10.1016/j.vetmic.2009.07.021 19854008

[65] Thanawongnuwech R, Thacker B, Halbur P, Thacker EL (2004) Increased production of proinflammatory cytokines following infection with porcine reproductive and respiratory syndrome virus and Mycoplasma hyopneumoniae. Clin Diagn Lab Immunol 11: 901–908. 15358650 10.1128/CDLI.11.5.901-908.2004 PMC515260

[66] Qiao S, Feng L, Bao D, Guo J, Wan B, Xiao Z, et al (2011) Porcine reproductive and respiratory syndrome virus and bacterial endotoxin act in synergy to amplify the inflammatory response of infected macrophages. Vet Microbiol 149: 213–220. 10.1016/j.vetmic.2010.11.006 21129861

[67] Van Gucht S, Labarque G, Van Reeth K (2004) The combination of PRRS virus and bacterial endotoxin as a model for multifactorial respiratory disease in pigs. Vet Immunol Immunopathol 102: 165–178. 15507303 10.1016/j.vetimm.2004.09.006 PMC7112634

[68] van Gucht S, van Reeth K, Pensaert M (2003) Interaction between porcine reproductive-respiratory syndrome virus and bacterial endotoxin in the lungs of pigs: potentiation of cytokine production and respiratory disease. J Clin Microbiol 41: 960–966. 12624016 10.1128/JCM.41.3.960-966.2003 PMC150282

