# Supplementary material for: Transcriptional Analysis of PRRSV-Infected Porcine Dendritic Cell Response to Streptococcus suis Infection Reveals Up-Regulation of Inflammatory-Related Genes Expression
Source: PLoS One. 2016 May 23;11(5):e0156019. doi: 10.1371/journal.pone.0156019 (PMC4877111; doi:10.1371/journal.pone.0156019)
Supplement: S1 Table — (DOCX) [file pone.0156019.s001.docx]

**Supplemental Table S1**: Genes upregulated greater than two-fold in porcine BMDCs after infection by PRRSV, *S. suis*, or co-infected with both pathogens for 12 h, compared to mock-infected cells

| **Genebank ID** | **Gene** | **Gene description** | **PRRSV** | ***S. suis*** | **Co-infection** |
| --- | --- | --- | --- | --- | --- |
| **Cytokines, chemokines, and related receptors** | | | | | |
| NM_213876 | *Amcf2* | Alveolar macrophage-derived chemotactic factor-II | 1.9 | **10.2** | **7.5** |
| NM_214214 | *Ccl2* | Chemokine (C-C motif) ligand 2 | 1.7 | **2.3** | **2.3** |
| NM_213779 | *Ccl4* | Chemokine (C-C motif) ligand 4 | **3.8** | **11.4** | **12.7** |
| AK233548 | *Ccl5* | Chemokine (C-C motif) ligand 5 | **19.1** | **4.7** | **30.7** |
| AK391248 | *Ccl8* | Chemokine (C-C motif) ligand 8 | 1.9 | **5.5** | **4.8** |
| NM_001256775 | *Ccl14* | Chemokine (C-C motif) ligand 14 | **2.2** | **7.7** | **10.9** |
| ENSSSCT00000023793 | *Ccl17* | Chemokine (C-C motif) ligand 17 | 1.4 | 1.7 | **2.1** |
| NM_001024589 | *Ccl20* | Chemokine (C-C motif) ligand 20 | **6.5** | **37.3** | **47.5** |
| NM_001009579 | *Ccl3l1* | Chemokine (C-C motif) ligand 3-like 1 | **2.2** | **6.6** | **7.3** |
| NM_214118 | *Csf2* | Colony stimulating factor 2 | **3.1** | **25.1** | **40.2** |
| NM_213842 | *Csf3* | Colony stimulating factor 3 | **2.0** | **10.7** | **10.2** |
| NM_001001861 | *Cxcl2* | Chemokine (C-X-C motif) ligand 2 | 1.9 | **10.3** | **9.2** |
| NM_214393 | *Ifna1* | Interferon, alpha 1 | **16.9** | **2.2** | **11.3** |
| NM_001166319 | *Ifna4* | Interferon-alpha-4 | **12.7** | 1.7 | **11.3** |
| NM_001003923 | *Ifnb1* | Interferon, beta 1, fibroblast | **300.9** | **10.9** | **308.7** |
| NM_001142837 | IFNλ1 | Interferon lambda 1, (Interleukin 29) | **2.0** | 1.2 | **2.2** |
| NM_214029 | *Il1a* | Interleukin 1, alpha | **3.1** | **13.5** | **14.0** |
| NM_001005149 | *IL1b* | Interleukin-1 beta | 1.5 | **22.2** | **23.5** |
| NM_214399 | *Il6* | Interleukin 6 | **3.2** | **36.3** | **45.1** |
| NM_001146128 | *Il7r* | Interleukin 7 receptor | **10.0** | **2.5** | **5.1** |
| X61151 | *Il8* | Interleukin 8 | 1.5 | **6.6** | **7.0** |
| AK235600 | *Il10rb* | Interleukin 10 receptor, beta | 1.1 | **2.1** | 1.8 |
| NM_213993 | *Il12a* | Interleukin 12A | **3.1** | **3.5** | **7.6** |
| NM_214013 | *Il12b* | Interleukin 12B | 1.9 | **3.3** | **5.7** |
| NM_214390 | *Il15* | Interleukin 15 | **3.1** | **2.1** | **4.9** |
| NM_213997 | *Il18* | Interleukin 18 | 0.8 | **2.5** | 1.6 |
| NM_001130236 | *Il23a* | Interleukin 23, alpha subunit p19 | 1.8 | **26.4** | **29.2** |
| NM_001007520 | *Il27* | Interleukin 27 | **2.5** | **7.3** | **7.9** |
| NM_214453 | *Lta* | Lymphotoxin alpha | 1.4 | **2.3** | **3.8** |
| NM_214321 | *Ptgs2* | Cyclooxygenase 2 | **6.5** | **5.3** | **11.0** |
| NM_214022 | *Tnf* | Tumor necrosis factor | **8.6** | **26.5** | **45.1** |
| NM_001267890 | *Tnfaip3* | Tumor necrosis factor, alpha-induced protein 3 | **2.1** | **2.7** | **3.6** |
| NM_001159607 | *Tnfaip6* | Tumor necrosis factor, alpha-induced protein 6 | 1.9 | **6.1** | **5.0** |
| NM_001025219 | *Tnfrsf8* | Tumor necrosis factor receptor superfamily, member 8 | 0.9 | **2.4** | **2.3** |
| AY426335 | *Tnfsf4* | Tumor necrosis factor (ligand) superfamily, member 4 | 1.7 | 1.0 | **2.1** |
| NM_001024696 | *Tnfsf10* | Tumor necrosis factor superfamily, member 10 | **5.9** | 1.8 | **4.6** |
| **Host defense** | | | | | |
| NM_001159307 | *Bpi* | Bactericidal/permeability-increasing protein | **2.0** | 0.8 | 1.9 |
| AF319661 | *Ddx58* | RIG-I | **4.5** | 1.7 | **4.8** |
| NM_001128473 | *Gbp1* | Guanylate binding protein 1 | **3.3** | 1.6 | **3.4** |
| NM_001143712 | *Gzmm* | Granzyme M | **4.3** | 1.3 | **7.2** |
| NM_001100194 | *Ifih1* | Interferon induced with helicase C domain 1 | **4.5** | **2.3** | **4.5** |
| NM_001244363 | *Ifit1* | Interferon-induced protein with tetratricopeptide repeats 1 | **9.2** | **2.3** | **8.3** |
| AK230663 | *Ifit2* | Interferon-induced protein with tetratricopeptide repeats 2 | **9.2** | **2.0** | **7.0** |
| NM_001204395 | *Ifit3* | Interferon-induced protein with tetratricopeptide repeats 3 | **5.5** | 1.8 | **4.4** |
| AK343937 | *Irg1* | Immunoresponsive 1 homolog | 1.7 | **12.7** | **7.8** |
| NM_213817 | *Irg6* | Inflammatory response protein 6 | **8.9** | **2.4** | **8.1** |
| XM_001926559 | *Jag1* | Jagged 1 | **3.2** | **7.5** | **11.1** |
| NM_214061 | *Mx1* | Myxovirus resistance 1 | **4.6** | 1.6 | **4.2** |
| AK345001 | *Mx2* | Myxovirus resistance 2 | **5.8** | **2.3** | **5.1** |
| NM_001256770 | *Nlrp3* | NLR family, pyrin domain containing 3 | 1.6 | **2.9** | **3.9** |
| NM_001105295 | *Nod2* | Nucleotide-binding oligomerization domain containing 2 | **2.4** | 1.9 | **3.1** |
| NM_001031796 | *Oas2* | 2'-5'-oligoadenylate synthetase 2 | **3.5** | 1.3 | **3.3** |
| NM_001244503 | *Pik3ap1* | Phosphoinositide-3-kinase adaptor protein 1 | 1.6 | 1.9 | **2.2** |
| NM_213851 | *Pik3r5* | Phosphoinositide-3-kinase, regulatory subunit 5 | 1.2 | **2.2** | **2.0** |
| ENSSSCT00000029122 | *Samd9* | Sterile alpha motif domain containing 9 | **2.2** | 1.7 | **2.1** |
| AK389803 | *Samsn1* | SAM domain, SH3 domain and nuclear localization signals 1 | 1.6 | **2.4** | **2.4** |
| ENSSSCT00000010505 | *Sema4d* | Sema domain, 4D | **2.1** | 0.9 | **2.3** |
| ENSSSCT00000004481 | *Tagap* | T-cell activation RhoGTPase activating protein | **2.6** | 0.7 | **2.3** |
| NM_001044581 | *Tap1* | Transporter 1, ATP-binding cassette, sub-family B (MDR/TAP) | **2.4** | 1.1 | **2.4** |
| AK238718 | *Trim21* | Tripartite motif containing 21 | **3.0** | 1.7 | **3.3** |
| **Surface receptor molecule and antigen presentation** | | | | | |
| NM_214194 | *Cd40* | CD40 molecule | 1.1 | **2.0** | 1.8 |
| NM_214091 | *Cd69* | CD69 molecule | **3.3** | **3.5** | **6.1** |
| NM_214087 | *Cd80* | CD80 molecule | 1.5 | **2.4** | **3.1** |
| NM_001025221 | *Cd274* | CD274 molecule | **3.2** | **3.8** | **5.7** |
| NM_001244418 | *Dll4* | Delta-like 4 | 1.2 | 1.2 | **3.7** |
| NM_213839 | *Fas* | Fas (TNF receptor superfamily, member 6) | 1.2 | **2.1** | **2.6** |
| AK237750 | *Gpr4* | G protein-coupled receptor 4 | 1.5 | **3.6** | **4.2** |
| **Cytokine signaling** | | | | | |
| AK392182 | *Ass1* | Argininosuccinate synthase 1 | **3.2** | 1.2 | **2.5** |
| ENSSSCT00000005115 | *Dmxl2* | Dmx-like 2 | 1.0 | **3.0** | **2.2** |
| NM_001244430 | *Dnajb1* | DnaJ (Hsp40) homolog, subfamily B, member 1 | **2.5** | 0.9 | **2.3** |
| NM_001097413 | *Irf1* | Interferon regulatory factor 1 | **3.0** | **2.8** | **4.9** |
| ENSSSCT00000005425 | *Malt1* | Mucosa associated lymphoid tissue lymphoma translocation gene 1 | 1.7 | 1.7 | **2.6** |
| NM_214319 | *Pkr* | Double stranded RNA-dependent protein kinase | **2.3** | 1.3 | **2.3** |
| ENSSSCT00000018432 | *Plk2* | Polo-like kinase 2 | 1.8 | **2.3** | **2.4** |
| ENSSSCT00000026377 | *Rasgef1b* | RasGEF domain family, member 1B | 1.8 | 1.5 | **2.3** |
| NM_001113017 | *Rgs16* | Regulator of G-protein signaling 16 | **2.3** | **4.0** | **9.2** |
| NM_001123189 | *Rhob* | Ras homolog family member B | **2.3** | 1.1 | **2.1** |
| NM_001123196 | *Socs3* | Suppressor of cytokine signaling 3 | **2.6** | **4.5** | **5.1** |
| NM_001197305 | *Stat4* | Signal transducer and activator of transcription 4 | 1.9 | 1.9 | **2.9** |
| AK237182 | *Steap4* | STEAP family member 4 | **2.5** | **9.5** | **9.6** |
| ENSSSCT00000033675 | *Tank* | TRAF family member-associated NFKB activator | 1.3 | 1.7 | **2.0** |
| ENSSSCT00000009650 | *Tec* | Tec protein tyrosine kinase | 1.8 | 1.6 | **2.2** |
| ENSSSCT00000033618 | *Trim36* | Tripartite motif containing 36 | 1.2 | **2.5** | **2.8** |
| **Cell adhesion and migration** | | | | | |
| NM_001001258 | *Atp4b* | Atpase, H+/K+ exchanging, beta polypeptide | 1.5 | 1.2 | **4.0** |
| EV991052 | *Bcr* | Breakpoint cluster region | **8.0** | **4.9** | **10.6** |
| AK349487 | *Capn6* | Calpain 6 | **3.3** | 0.9 | **3.0** |
| NM_001109945 | *Cdh13* | Cadherin 13 | **2.2** | 1.5 | **2.2** |
| ENSSSCT00000012469 | *Cdhr4* | Cadherin-related family member 4 | **2.7** | 1.0 | **2.1** |
| NM_213816 | *Icam1* | Intercellular adhesion molecule-1 | 1.2 | **4.1** | **3.9** |
| NM_001083932 | *Itgav* | Integrin, alpha V | 1.3 | 1.7 | **2.3** |
| CN159825 | *Myhc* | Myosin heavy chain | **4.5** | 0.9 | **4.0** |
| CX065926 | *Nuak2* | NUAK family, SNF1-like kinase, 2 | **10.9** | 1.3 | **11.8** |
| NM_001244188 | *Peak1* | NKF3 kinase family member | **2.7** | 1.4 | **3.6** |
| NM_214284 | *Sdc4* | Syndecan 4 | 1.2 | **2.5** | **2.3** |
| BW999788 | *Sptb* | Spectrin, beta | **7.2** | 1.3 | **6.7** |
| **Transcriptional and translational regulation** | | | | | |
| NM_001243031 | *Cc2d1a* | Coiled-coil and C2 domain containing 1A | **5.8** | 1.3 | **5.4** |
| NM_213880 | *Cjun* | C-JUN protein | 1.9 | 1.3 | **2.2** |
| NM_001099929 | *Creb1* | cAMP responsive element binding protein 1 | **2.1** | 1.4 | **2.6** |
| BX675204 | *Egr1* | Early growth response 1 | **4.8** | 1.5 | **6.3** |
| DQ355795 | *Egr3* | Early growth response 3 | **2.8** | 1.7 | **3.1** |
| NM_001194975 | *Eif2c2* | Eukaryotic translation initiation factor 2C, 2 | 1.5 | 1.7 | **2.1** |
| AK232994 | *Fos* | FBJ murine osteosarcoma viral oncogene homolog | **5.0** | 0.6 | **2.7** |
| NM_001097489 | *Klf5* | Kruppel-like factor 5 (intestinal) | 1.5 | **2.6** | **2.6** |
| NM_001134348 | *Klf14* | Kruppel-like factor 14 | 1.8 | 1.4 | **2.2** |
| AY609599 | *Nfkbia* | Nuclear factor of kappa light polypeptide gene enhancer in B-cells inhibitor, alpha | **2.3** | **3.1** | **4.6** |
| NM_001048232 | *Nfkb1* | Nuclear factor of kappa light polypeptide gene enhancer in B-cells 1 | **2.2** | **2.3** | **3.8** |
| NM_001190276 | *Nr4a2* | Nuclear receptor subfamily 4, group A, member 2 | **3.4** | 1.0 | **2.5** |
| NM_001097499 | *Nr6a1* | Nuclear receptor subfamily 6, group A, member 1 | 1.8 | 1.5 | **3.5** |
| NM_214040 | *Oct2* | POU-domain protein | 1.2 | **2.8** | **2.3** |
| AK347698 | *Parp11* | Poly (ADP-ribose) polymerase family, member 11 | **3.1** | 1.3 | **2.4** |
| ENSSSCT00000017966 | *Parp12* | Poly (ADP-ribose) polymerase family, member 12 | **2.5** | 1.0 | **2.3** |
| AK398616 | *Parp14* | Poly (ADP-ribose) polymerase family, member 14 | **3.2** | 1.7 | **3.1** |
| NM_001244178 | *Pax6* | Paired box 6 | **3.6** | 1.0 | **3.7** |
| AY653213 | *Pax7* | Paired box 7 | **11.6** | 1.3 | **10.8** |
| XM_001925350 | *Prdm1* | PR domain containing 1 | **6.0** | **2.2** | **7.7** |
| NM_001044528 | *Rnf4* | Ring finger protein 4 | **2.0** | 0.9 | **2.2** |
| NM_001038006 | *Rnf19* | Ring finger protein 19 | 1.5 | 1.9 | **2.2** |
| NM_214137 | *Smad3* | SMAD family member 3 | **2.2** | 0.8 | 1.2 |
| ENSSSCT00000031400 | *Supt16h* | Suppressor of Ty 16 homolog | 1.2 | 1.9 | **2.3** |
| AK347963 | *Tcf7l2* | Transcription factor 7-like 2 | 1.7 | **2.1** | **2.8** |
| ENSSSCT00000008047 | *Top1* | Topoisomerase I | 1.8 | 1.3 | **2.2** |
| NM_001244241 | *Wtap* | Wilms tumor 1 associated protein | 1.6 | 1.7 | **2.2** |
| NM_001123216 | *Zbp1* | Z-DNA binding protein 1 | **2.7** | 1.3 | **2.4** |
| ENSSSCT00000004521 | *Zbtb2* | Zinc finger and BTB domain containing 2 | 1.9 | 0.8 | **2.0** |
| ENSSSCT00000006749 | *Zbtb10* | Zinc finger and BTB domain containing 10 | 1.4 | **2.0** | **2.1** |
| ENSSSCT00000015562 | *Znf608* | Zinc finger protein 608 | 1.8 | 1.7 | **2.5** |
| ENSSSCT00000007359 | *Znf697* | Zinc finger protein 697 | 1.4 | **2.2** | 1.9 |
| **Vascular homeostasis, wound healing** | | | | | |
| NM_001144843 | *Adamts1* | ADAM metallopeptidase with thrombospondin type 1 motif, 1 | **3.8** | **2.5** | **5.6** |
| NM_214107 | *Adm* | Adrenomedullin | 1.7 | **5.0** | **5.3** |
| NM_214376 | *Areg* | Amphiregulin | **9.2** | **6.8** | **25.2** |
| NM_001039745 | *Bmpr1b* | Bone morphogenetic protein receptor, type IB | **3.3** | 1.1 | **3.8** |
| NM_213882 | *Edn1* | Endothelin 1 | **8.6** | **5.4** | **17.4** |
| NM_001098582 | *Edn3* | Endothelin 3 | **8.1** | **4.7** | **12.1** |
| NM_213785 | *F3* | Coagulation factor III | 1.8 | **9.2** | **13.7** |
| NM_214299 | *Hbegf* | Heparin-binding EGF-like growth factor | **3.5** | **3.0** | **5.0** |
| NM_001166308 | *Mmp3* | Matrix metallopeptidase 3 | 1.5 | **6.9** | **8.4** |
| XM_001927177 | *Mmrn2* | Multimerin 2 | 1.1 | **6.7** | **4.3** |
| NM_001008482 | *Nppc* | Natriuretic peptide C | 1.5 | 2.1 | **3.1** |
| NM_001244459 | *Sgk1* | Serum/glucocorticoid regulated kinase 1 | **2.2** | 1.7 | **3.1** |
| NM_214084 | *Vegfa* | Vascular endothelial growth factor A | **4.4** | **3.1** | **4.4** |
| **Apoptosis, cell cycle regulation, and oncogenesis** | | | | | |
| ENSSSCT00000001993 | *Bcl2a1* | BCL2-related protein A1 | 1.0 | **2.1** | 1.9 |
| NM_001099936 | *Btg1* | B-cell translocation gene 1, anti-proliferative | 1.3 | **2.0** | 1.9 |
| NM_001097505 | *Btg2* | BTG family, member 2 | 1.2 | **4.7** | **4.3** |
| NM_001097517 | *Btg3* | BTG family, member 3 | 1.2 | **2.3** | **2.5** |
| NM_001161640 | *Casp10* | Caspase 10 | **2.0** | **3.0** | **3.4** |
| NM_001001628 | *Cflip* | Cellular FLICE-like inhibitory protein | 1.3 | **3.1** | **3.3** |
| NM_001005728 | *Hig2* | Hypoxia-inducible protein | 1.1 | **4.7** | **4.0** |
| NM_213766 | *Hsp70.2* | Heat shock protein 70.2 | **3.6** | 1.2 | **3.2** |
| NM_001097504 | *Hsph1* | Heat shock 105kda/110kda protein 1 | **3.3** | 1.1 | **3.2** |
| NM_214189 | *Inha* | Inhibin, alpha | **4.1** | 1.0 | **3.7** |
| NM_214247 | *Nor1* | Neuron-derived orphan receptor-1 alpha | **3.9** | 1.7 | **4.3** |
| XM_001929598 | *Pim1* | Pim-1 oncogene | 1.4 | **3.2** | **3.3** |
| AK392629 | *Plk3* | Polo-like kinase 3 | **2.6** | **2.3** | **3.9** |
| NM_214147 | *Pmaip1* | Phorbol-12-myristate-13-acetate-induced protein 1 | **3.4** | **2.7** | **4.8** |
| ENSSSCT00000013823 | *Stag2* | Stromal antigen 2 | 1.8 | 1.2 | **2.1** |
| **Complement cascade** | | | | | |
| NM_001101824 | *Cfb* | Complement factor B | 1.1 | **2.4** | 1.8 |
| **Lipid Metabolism** | | | | | |
| ENSSSCT00000011416 | *A1cf* | APOBEC1 complementation factor | **2.1** | 1.0 | **2.5** |
| NM_214246 | *Ces1* | Carboxylesterase 1 | 1.6 | **2.1** | 1.8 |
| NM_001160080 | *Dgat2* | Diacylglycerol O-acyltransferase 2 | **2.6** | 0.9 | **2.6** |
| AK236400 | *Ldlr* | Low density lipoprotein receptor | 1.8 | **2.0** | **2.3** |
| NM_001243668 | *Paqr6* | Progestin and adipoQ receptor family member VI | **2.3** | 1.0 | **2.3** |
| NM_214052 | *Plcd4* | Phospholipase C, delta 4 | **2.4** | 1.2 | **2.2** |
| NM_001145222 | *Rbp7* | Retinol binding protein 7, cellular | 1.5 | 1.7 | **2.1** |
| **Biological and metabolic process** | | | | | |
| ENSSSCT00000011534 | *Abcc2* | ATP-binding cassette, sub-family C (CFTR/MRP), member 2 | **2.7** | 0.9 | **2.8** |
| AK398470 | *Abtb2* | Ankyrin repeat and BTB (POZ) domain containing 2 | **2.2** | **2.8** | **4.3** |
| NM_001167629 | *Acsl1* | Acyl-coa synthetase long-chain family member 1 | 1.4 | **3.3** | **2.4** |
| ENSSSCT00000011015 | *Adora2a* | Adenosine A2a receptor | 1.3 | **2.7** | **2.9** |
| NM_001044597 | *Ak3l1* | Adenylate kinase 3-like 1 | 0.9 | **2.2** | 1.6 |
| NM_001123076 | *Ampd1* | Adenosine monophosphate deaminase 1 | **2.9** | 0.9 | 1.7 |
| NM_214048 | *Arg1* | Arginase, liver | 1.0 | **2.2** | 1.7 |
| ENSSSCT00000002508 | *Arrdc4* | Arrestin domain containing 4 | 1.5 | 1.8 | **2.7** |
| ENSSSCT00000006270 | *Ass1* | Argininosuccinate synthase 1 | **3.2** | 1.2 | **2.5** |
| ENSSSCT00000006901 | *Atp1b1* | ATPase, Na+/K+ transporting, beta 1 polypeptide | 1.0 | **2.0** | 1.4 |
| NM_001195399 | *Bmp2* | Bone morphogenetic protein 2 | 1.6 | 1.5 | **2.7** |
| NM_001243920 | *Ca4* | Carbonic anhydrase IV | 1.6 | **3.2** | **3.8** |
| ENSSSCT00000017131 | *Clasp1* | Cytoplasmic linker associated protein 1 | 1.8 | 1.9 | **2.3** |
| NM_001113015 | *Cmah* | Cytidine monophosphate-N-acetylneuraminic acid hydroxylase | 1.8 | 1.5 | **2.4** |
| NM_001004026 | *Csn3* | Casein kappa | **2.9** | 1.5 | **2.2** |
| AK349562 | *Csrnp1* | Cysteine-serine-rich nuclear protein 1 | **2.5** | 1.5 | **3.7** |
| NM_001129970 | *Cycs* | Cytochrome C, somatic | 1.2 | **2.2** | 1.1 |
| NM_214339 | *Dnaja4* | DnaJ (Hsp40) homolog, subfamily A, member 4 | **3.0** | 0.9 | **2.9** |
| EW668995 | *Dppa5* | Developmental pluripotency associated 5 | 1.2 | **2.1** | 1.7 |
| XM_001929319 | *Entpd7* | Ectonucleoside triphosphate diphosphohydrolase 7 | 1.9 | **2.2** | **2.7** |
| NM_001190218 | *Fam177a1* | Family with sequence similarity 177, member A1 | 0.9 | **2.3** | 1.6 |
| NM_001003662 | *Fst* | Follistatin | 1.3 | **2.0** | 1.9 |
| ENSSSCT00000006767 | *Gdap1* | Ganglioside induced differentiation associated protein 1 | **2.9** | 1.4 | 1.4 |
| Z80109 | *Gem* | GTP binding protein overexpressed in skeletal muscle | **2.5** | **2.7** | **4.8** |
| NM_001122987 | *Hk2* | Hexokinase 2 | 1.1 | 1.8 | **2.2** |
| ENSSSCT00000012468 | *Ip6k1* | Inositol hexakisphosphate kinase 1 | 1.8 | 1.1 | **2.1** |
| NM_001110418 | *Kcna2* | Potassium voltage-gated channel, shaker-related subfamily, member 2 | **2.5** | 1.1 | **2.1** |
| ENSSSCT00000007068 | *Kirrel* | Kin of IRRE like | **2.7** | 1.3 | **2.8** |
| AY609400 | *Lysmd2* | Lysm, putative peptidoglycan-binding, domain containing 2 | 1.6 | **3.9** | **4.2** |
| ENSSSCT00000030573 | *Mb21d1* | Mab-21 domain containing 1 | **2.4** | 1.3 | **2.2** |
| NM_001258293 | *Mesdc1* | Mesoderm development candidate 1 | 1.2 | **2.4** | **2.1** |
| AK232361 | *Mfsd2a* | Major facilitator superfamily domain containing 2A | **2.3** | **2.3** | **5.7** |
| NM_001244607 | *Mllt11* | Myeloid/lymphoid or mixed-lineage leukemia (trithorax homolog, Drosophila); translocated to, 11 | 0.9 | **2.5** | **2.0** |
| ENSSSCT00000003142 | *N4bp1* | NEDD4 binding protein 1 | 1.7 | 1.9 | **2.1** |
| NM_001031793 | *NamptT* | Nicotinamide phosphoribosyltransferase | 1.4 | **2.3** | **2.2** |
| ENSSSCT00000012280 | *Ngly1* | N-glycanase 1 | 1.6 | 1.2 | **2.0** |
| NM_214247 | *Nor1* | Neuron-derived orphan receptor-1 alpha | **2.1** | 1.8 | **2.6** |
| NM_213989 | *Oct1* | Organic cation transporter 1 | **2.5** | 1.2 | **2.0** |
| AK348181 | *Pla2g3* | Phospholipase A2, group III | **12.1** | 0.9 | **10.8** |
| NM_214266 | *Prkaa2* | Protein kinase, AMP-activated, alpha 2 catalytic subunit | 0.9 | **2.4** | 1.8 |
| NM_001044577 | *Rhcg* | Rh family, C glycoprotein | 0.8 | **6.3** | **2.3** |
| NM_214296 | *Rnd3* | Rho family GTPase 3 | **2.2** | **2.1** | **2.6** |
| AK347139 | *Rora* | RAR-related orphan receptor A | 1.4 | **2.1** | **2.5** |
| AK233471 | *Sds* | Serine dehydratase | 1.5 | **4.7** | **4.2** |
| NM_001102679 | *Sftpb* | Surfactant protein B | **2.7** | 1.0 | **2.2** |
| X17058 | *Slc2a1* | Solute carrier family 2, member 1 | 1.2 | 1.9 | **2.5** |
| AK389853 | *Slc2a3* | Solute carrier family 2, member 3 | 1.8 | **4.1** | **4.8** |
| NM_001128433 | *Slc2a4* | Solute carrier family 2, member 4 | 1.2 | 1.7 | **2.5** |
| XM_001929323 | *Slc6a17* | Solute carrier family 6, member 17 | **4.4** | 1.1 | **3.8** |
| AF123280 | *Slc9a3* | Solute carrier family 9, subfamily A, member 3 | **17.0** | 1.4 | **14.8** |
| NM_001128440 | *Slc11a2* | Solute carrier family 11, member 2 | 1.0 | **3.3** | **2.4** |
| NM_001164514 | *Slc25a19* | Solute carrier family 25, member 19 | 1.3 | **2.5** | **2.1** |
| NM_001164510 | *Slc25a25* | solute carrier family 25, member 25 | **2.7** | 0.8 | **2.3** |
| DQ529289 | *Slc35d1* | Solute carrier family 35, member D1 | 1.5 | 1.7 | **2.1** |
| XM_001925697 | *Slc39a14* | Solute carrier family 39, member 14 | 1.7 | **4.9** | **4.6** |
| ENSSSCT00000031745 | *Sod2* | Superoxide dismutase 2 | 0.7 | **2.9** | 1.6 |
| NM_213755 | *Star* | Steroidogenic acute regulatory protein | **5.2** | 1.0 | **4.1** |
| ENSSSCT00000001964 | *Tbc1d2B* | TBC1 domain family, member 2B | 1.4 | 2.1 | **2.2** |
| NM_001145754 | *Tcap* | Titin-cap (telethonin) | **2.3** | 1.0 | 1.8 |
| ENSSSCT00000036171 | *Tg* | Thyroglobulin | 1.3 | 1.7 | **2.2** |
| AK343870 | *Tiparp* | TCDD-inducible poly(ADP-ribose) polymerase | **2.0** | 1.4 | **2.6** |
| NM_001243294 | *Tmod4* | Tropomodulin 4 | **2.4** | 1.2 | 1.9 |
| NM_214049 | *Ucp3* | Uncoupling protein 3 | **3.1** | 1.3 | **2.2** |
| NM_213826 | *Usp18* | Ubiquitin specific peptidase 18 | **4.4** | 1.9 | **4.3** |
| NM_001123215 | *Wap-1* | Elafin | 1.9 | 0.9 | **2.5** |
