# Supplementary material for: Transcriptional Analysis of PRRSV-Infected Porcine Dendritic Cell Response to Streptococcus suis Infection Reveals Up-Regulation of Inflammatory-Related Genes Expression
Source: PLoS One. 2016 May 23;11(5):e0156019. doi: 10.1371/journal.pone.0156019 (PMC4877111; doi:10.1371/journal.pone.0156019)
Supplement: S2 Table — (DOCX) [file pone.0156019.s002.docx]

**Supplemental Table S2**: Genes downregulated greater than two-fold in porcine BMDCs after infection by PRRSV, *S. suis*, or co-infected with both pathogens for 12 h, compared to mock-infected cells

| **Genebank ID** | **Gene** | **Gene description** | **PRRSV** | ***S. suis*** | **Co-infection** |
| --- | --- | --- | --- | --- | --- |
| **Cytokines, chemokines, and related receptors** | | | | | |
| NM_001112690 | *April* | A proliferation-inducing ligand | **2.0** | 1.3 | **2.3** |
| NM_001123100 | *Cmklr1* | Chemokine-like receptor 1 | 1.4 | **2.3** | **2.1** |
| **Host defense** | | | | | |
| NM_001030534 | *Tlr10* | Toll-like receptor 10 | **2.3** | 1.9 | **4.0** |
| ENSSSCT00000010983 | *Pik3ip1* | phosphoinositide-3-kinase interacting protein 1 | 1.8 | **2.7** | **2.7** |
| ENSSSCT00000004324 | *Pik3r3* | Phosphoinositide-3-kinase, regulatory subunit 3 (gamma) | 1.6 | 1.9 | **2.5** |
| **Surface receptor molecule and antigen presentation** | | | | | |
| NM_001128436 | *Adrb2* | Adrenoceptor beta 2, surface | 1.1 | **4.8** | **3.2** |
| NM_001129972 | *Cd209* | CD209 molecule | **3.3** | 1.0 | **3.4** |
| **Cytokine signaling** | | | | | |
| ENSSSCT00000022796 | *Dixdc1* | DIX domain containing 1 | 1.5 | 1.8 | **2.2** |
| AK237720 | *Ly9* | Lymphocyte antigen 9 | 1.6 | 1.6 | **2.2** |
| AF339021 | *Plk1* | Polo-like kinase 1 | 1.1 | **2.1** | **2.1** |
| **Cell adhesion and migration** | | | | | |
| NM_001190244 | *Gpr1* | G protein-coupled receptor 1 | **2.6** | **2.5** | **3.6** |
| AK232986 | *Itih3* | Inter-alpha-trypsin inhibitor heavy chain 3 | **2.2** | 1.5 | **2.2** |
| NM_213942 | *C4bpa* | Complement component 4 binding protein, alpha | **3.1** | 1.5 | **3.5** |
| NM_001244215 | *C5ar1* | Complement component 5a receptor 1 | 1.3 | **2.6** | **2.1** |
| **Transcriptional and translational regulation** | | | | | |
| ENSSSCT00000011643 | *Dclre1a* | DNA cross-link repair 1A | 1.3 | 1.5 | **2.0** |
| AK233254 | *Deptor* | DEP domain containing MTOR-interacting protein | **2.3** | **3.0** | **3.5** |
| NM_001244497 | *Fbxo21* | F-box protein 21 | 1.9 | 1.4 | **2.5** |
| NM_213946 | *Fhl3* | Four and a half LIM domains 3 | 1.3 | **2.0** | 1.9 |
| NM_001244579 | *Hhex* | Hematopoietically expressed homeobox | 1.7 | 1.7 | **2.4** |
| AK351856 | *Hjurp* | Holliday junction recognition protein | 1.1 | 1.8 | **3.0** |
| NM_001134344 | *Klf10* | Kruppel-like factor 10 | 1.0 | **2.1** | 1.6 |
| GQ274321 | *Klf13* | Kruppel-like factor 13 | 1.1 | **2.1** | 1.5 |
| EF486522 | *Mef2c* | Myocyte enhancer factor 2C | 1.6 | **2.6** | **3.0** |
| ENSSSCT00000012519 | *Phf7* | PHD finger protein 7 | 1.5 | **2.2** | 1.3 |
| ENSSSCT00000009936 | *Phf17* | PHD finger protein 17 | 1.6 | 1.7 | **2.2** |
| AK389505 | *Pik3ip1* | Phosphoinositide-3-kinase interacting protein 1 | 1.6 | **3.2** | **2.1** |
| XM_003480446 | *Smad6* | SMAD family member 6 | 1.5 | **3.3** | **2.8** |
| ENSSSCT00000010254 | *Smad9* | SMAD family member 9 | 1.9 | 1.5 | **2.2** |
| ENSSSCT00000025296 | *Snapc5* | Small nuclear RNA activating complex, polypeptide 5 | 1.2 | **2.0** | **2.4** |
| ENSSSCT00000009350 | *Znf512* | Zinc finger protein 512 | 1.4 | 1.6 | **2.1** |
| ENSSSCT00000010669 | *Znf605* | Zinc finger protein 605 | 1.3 | 1.9 | **2.2** |
| **Apoptosis, cell cycle regulation, and oncogenesis** | | | | | |
| NM_001123094 | *Cdc20* | Cell division cycle 20 homolog | 1.1 | 1.9 | **2.4** |
| ENSSSCT00000022716 | *Cep85* | Centrosomal protein 85kDa | 1.5 | 1.7 | **2.3** |
| ENSSSCT00000010370 | *Dis3* | DIS3 mitotic control homolog | 1.5 | 1.5 | **2.1** |
| NM_001243602 | *Id3* | Inhibitor of DNA binding 3 | 1.3 | **2.5** | **3.6** |
| AK233842 | *Kifc1* | Kinesin family member C1 | 1.0 | **2.0** | 1.1 |
| ENSSSCT00000016997 | *Nek2* | NIMA (never in mitosis gene a)-related kinase 2 | 1.0 | **2.6** | 1.8 |
| XM_003355266 | *Psrc1* | Proline/serine-rich coiled-coil 1 | 1.3 | **2.1** | **2.0** |
| NM_001123174 | *Rab22a* | RAB22A, member RAS oncogene family | 1.8 | 1.4 | **2.9** |
| **Complement cascade** | | | | | |
| NM_213942 | *C4bpa* | Complement component 4 binding protein, alpha | **3.1** | 1.5 | **3.5** |
| NM_001244215 | *C5ar1* | Complement component 5a receptor 1 | 1.3 | **2.6** | **2.1** |
| **Lipid metabolism** | | | | | |
| ENSSSCT00000000097 | *Apobec3f* | Apolipoprotein B mRNA editing enzyme, catalytic polypeptide-like 3F | 1.6 | 1.7 | **2.3** |
| NM_001244565 | *Cln8* | Ceroid-lipofuscinosis, neuronal 8 | 1.5 | 1.9 | **2.0** |
| AK346249 | *Dhcr24* | 24-dehydrocholesterol reductase | 1.0 | **2.1** | 1.9 |
| ENSSSCT00000030171 | *Lpin1* | Lipin 1 | 1.3 | **2.1** | 1.9 |
| **Biological and metabolic process** | | | | | |
| ENSSSCT00000000762 | *Acrbp* | Acrosin binding protein | 1.7 | 1.5 | **2.2** |
| NM_001143695 | *Acss2* | Acyl-CoA synthetase short-chain family member 2 | **2.2** | **2.0** | **2.4** |
| ENSSSCT00000019305 | *Adap2* | ArfGAP with dual PH domains 2 | 1.3 | **2.2** | **2.0** |
| AK238941 | *Alg5* | Asparagine-linked glycosylation 5 | 1.5 | 1.7 | **2.1** |
| ENSSSCT00000003036 | *Ap1g1* | Adaptor-related protein complex 1, gamma 1 subunit | 1.9 | 1.1 | **2.1** |
| ENSSSCT00000010108 | *Arhgap24* | Rho GTPase activating protein 24 | 1.5 | 1.8 | **2.1** |
| AK394278 | *Atp5b* | ATP synthase, H+ transporting, mitochondrial F1 complex, beta polypeptide | 1.4 | 1.4 | **2.3** |
| NM_001044534 | *Cds1* | CDP-diacylglycerol synthase (phosphatidate cytidylyltransferase) 1 | 1.6 | 1.7 | **2.6** |
| ENSSSCT00000011587 | *Cnnm2* | Cyclin M2 | **2.0** | **2.0** | **2.9** |
| NM_001007517 | *Cox5b* | Mitochondrial cytochrome C oxidase subunit V b | **2.5** | 0.8 | **2.4** |
| ENSSSCT00000017364 | *Dcaf17* | DDB1 and CUL4 associated factor 17 | 1.2 | 1.4 | **2.2** |
| ENSSSCT00000011072 | *Dgcr2* | DiGeorge syndrome critical region gene 2 | 1.0 | **2.0** | 1.1 |
| ENSSSCT00000008013 | *Dsn1* | DSN1, MIND kinetochore complex component, homolog | 1.3 | **2.1** | **2.2** |
| NM_001145385 | *Dynlt1* | Dynein, light chain, Tctex-type 1 | 1.9 | 1.1 | **2.2** |
| AK234578 | *Fam96a* | Family with sequence similarity 96, member A | 1.6 | 1.3 | **2.1** |
| NM_213797 | *Fate1* | Fetal and adult testis expressed 1 | 1.4 | 1.2 | **2.1** |
| NM_001204852 | *Fpgt* | Fucose-1-phosphate guanylyltransferase | 1.5 | 1.8 | **2.7** |
| ENSSSCT00000014010 | *Fundc2* | FUN14 domain containing 2 | 1.1 | 1.7 | **2.0** |
| ENSSSCT00000000741 | *Gpr162* | G protein-coupled receptor 162 | 1.9 | **2.2** | **2.9** |
| AK345817 | *Ivd* | Isovaleryl-CoA dehydrogenase | 1.8 | 1.7 | **2.5** |
| NM_214093 | *Kcne3* | Potassium voltage-gated channel, Isk-related family, member 3 | 1.6 | **2.0** | 1.9 |
| ENSSSCT00000013264 | *Ofd1* | Oral-facial-digital syndrome 1 | 1.3 | **2.0** | 1.9 |
| ENSSSCT00000010760 | *P2rx7* | Purinergic receptor P2X, ligand-gated ion channel, 7 | 1.7 | 1.9 | **2.6** |
| AK348897 | *P2ry1* | Purinergic receptor P2Y, G-protein coupled, 1 | 1.8 | **2.5** | **3.1** |
| XM_001926022 | *Pank1* | Pantothenate kinase 1 | 1.3 | **2.0** | **2.1** |
| XM_001928961 | *Pnma1* | Paraneoplastic Ma antigen 1 | 1.6 | 1.6 | **2.3** |
| NM_001190183 | *Prmt6* | Protein arginine methyltransferase 6 | 1.2 | 1.6 | **2.0** |
| ENSSSCT00000010990 | *Prr14l* | Proline rich 14-like | **2.3** | 0.9 | **2.2** |
| NM_001097516 | *Sdhd* | Succinate dehydrogenase complex, subunit D | **2.3** | 1.1 | **2.1** |
| AK394625 | *Sgcb* | Sarcoglycan, beta | **2.0** | 1.3 | **2.4** |
| NM_001243383 | *Slc46a1* | Solute carrier family 46, member 1 | **2.0** | **2.0** | **3.2** |
| AK233023 | *Slc46a3* | Solute carrier family 46, member 3 | 1.7 | **2.0** | **2.5** |
| AK351365 | *Tdp1* | Tyrosyl-DNA phosphodiesterase 1 | 1.4 | **2.1** | 1.6 |
| ENSSSCT00000004109 | *Ttc39c* | Tetratricopeptide repeat domain 39C | 1.2 | 1.7 | **2.1** |
| ENSSSCT00000011229 | *Tysnd1* | Trypsin domain containing 1 | 1.1 | **2.1** | 1.6 |
