# Supplementary material for: Transcriptional Analysis of PRRSV-Infected Porcine Dendritic Cell Response to Streptococcus suis Infection Reveals Up-Regulation of Inflammatory-Related Genes Expression
Source: PLoS One. 2016 May 23;11(5):e0156019. doi: 10.1371/journal.pone.0156019 (PMC4877111; doi:10.1371/journal.pone.0156019)
Supplement: S3 Table — (DOCX) [file pone.0156019.s003.docx]

**Supplemental Table S3:** Genes upregulated greater than two-fold in porcine monocytes after infection by PRRSV, *S. suis*, or co-infected with both pathogens for 12 h, compared to mock-infected cells

| **Genebank ID** | **Gene** | **Gene description** | **PRRSV** | ***S. suis*** | **Co-infection** |
| --- | --- | --- | --- | --- | --- |
| **Cytokines, chemokines, and related receptors** | | | | | |
| AK233548 | *Ccl5* | Chemokine (C-C motif) ligand 5 | 1.2 | **3.0** | **2.7** |
| NM_001024589 | *Ccl20* | Chemokine (C-C motif) ligand 20 | 1.4 | **5.0** | **9.6** |
| NM_214118 | *Csf2* | Colony Stimulating Factor 2 | 0.8 | **6.3** | **9.1** |
| NM_213842 | *Csf3* | Colony stimulating factor 3 | 0.9 | **2.5** | **3.6** |
| NM_001003923 | *Ifnb1* | Interferon beta | **2.6** | 1.3 | **2.1** |
| NM_001005149 | *Il1b* | Interleukin-1 beta | 1.0 | 1.5 | **2.6** |
| NM_213835 | *Il2ra* | Interleukin 2 receptor, alpha | 1.0 | 1.9 | **2.6** |
| NM_001146128 | *Il7r* | Interleukin 7 receptor | 1.3 | **2.4** | **2.3** |
| NM_214041 | *Il10* | Interleukin 10 | 0.5 | **2.4** | 1.7 |
| NM_214013 | *Il12b* | Interleukin 12p40 | 0.8 | **19.4** | **25.8** |
| NM_214262 | *Il1rn* | Interleukin 1 receptor antagonist | 0.8 | **5.0** | **5.7** |
| NM_001130236 | *Il23a* | Interleukin 23 alpha subunit p19 | 0.8 | **10.1** | **16.5** |
| AK232305 | *Ptgs1* | Cyclooxygenase 1 | 1.2 | **3.3** | **3.8** |
| NM_214321 | *Ptgs2* | Cyclooxygenase 2 | 0.6 | **2.5** | **2.4** |
| NM_214023 | *Spp1* | Secreted phosphoprotein 1 | 1.6 | **2.3** | **3.8** |
| NM_214084 | *Vegfa* | Vascular endothelial growth factor A | 1.4 | 1.6 | **2.2** |
| **Host Defense** | | | | | |
| NM_214107 | *Adm* | Adrenomedullin | 1.3 | **2.1** | **2.4** |
| NM_214442 | *Pbd2* | Beta-defensin 2 | 0.8 | **2.7** | **2.1** |
| NM_001143709 | *Gzma* | Granzyme A | 1.3 | 1.7 | **2.0** |
| NM_001204395 | *Ifit3* | Interferon-induced protein with tetratricopeptide repeats 3 | **2.6** | 0.6 | 0.7 |
| NM_214303 | *Oas1* | 2'-5'-oligoadenylate synthetase 1, 40/46kDa | **2.8** | 0.3 | 0.6 |
| NM_001031796 | *Oas2* | 2'-5'-oligoadenylate synthetase 2, 69/71kDa | **2.1** | 0.9 | 1.1 |
| XM_001929523 | *Sh2b3* | SH2B adaptor protein 3 | 1.0 | **2.3** | **2.1** |
| **Surface receptor molecule and antigen presentation** | | | | | |
| NM_214194 | *Cd40* | CD40 molecule | 0.9 | 1.7 | **2.1** |
| NM_001243714 | *Cd48* | CD48 molecule | 0.8 | **2.0** | 1.9 |
| NM_214091 | *Cd69* | CD69 molecule | 1.0 | 1.9 | **2.0** |
| NM_214087 | *Cd80* | CD80 molecule | 1.1 | **2.4** | **2.7** |
| NM_213839 | *Fas* | Fas (TNF receptor superfamily, member 6) | 1.0 | **2.0** | **2.1** |
| **Cytokine signaling** | | | | | |
| NM_213766 | *Hsp70.2* | Heat shock protein 70.2 | 0.8 | **2.9** | **2.3** |
| NM_214204 | *Pkia* | Protein kinase inhibitor alpha | 1.0 | 1.6 | **2.3** |
| NM_001038631 | *Ptges* | Prostaglandin E synthase | 1.2 | **3.4** | **5.2** |
| NM_001113017 | *Rgs16* | Regulator of G-protein signaling 16 | 0.8 | **3.2** | **3.0** |
| NM_001197305 | *Stat4* | Signal transducer and activator of transcription 4 | 0.9 | **2.7** | **2.7** |
| **Cell adhesion and migration** | | | | | |
| AK349087 | *Cldn6* | Claudin 6 | **2.2** | **2.2** | 1.8 |
| ENSSSCT00000017611 | *Fn1* | Fibronectin 1 | **3.0** | 1.1 | **3.0** |
| ENSSSCT00000000314 | *Itga5* | Integrin, alpha 5 | 0.9 | **2.3** | **2.1** |
| NM_214192 | *Mmp2* | Matrix metallopeptidase 2 | 0.8 | 1.9 | **2.2** |
| NM_213891 | *Vcam1* | Vascular cell adhesion molecule 1 | **2.5** | **3.8** | **7.1** |
| **Transcriptional and translational regulation** | | | | | |
| EU669075 | Klf4 | Kruppel-like factor 4 | 1.1 | **2.1** | **2.2** |
| NM_001097489 | Klf5 | Kruppel-like factor 5 | 0.8 | **2.3** | **2.2** |
| AK240400 | Osr2 | Odd-skipped related 2 | **2.3** | 1.2 | 1.3 |
| **Vascular homeostasis, wound healing** | | | | | |
| NM_213882 | *Edn1* | Endothelin 1 | 0.7 | **5.2** | **4.1** |
| NM_001098582 | *Edn3* | Endothelin 3 | 0.9 | **4.1** | **3.1** |
| AK232765 | *Fn1* | Fibronectine 1 | **3.4** | 1.1 | **3.3** |
| NM_214054 | *Plat* | Plasminogen activator, tissue | 1.2 | **4.0** | **4.0** |
| AK230691 | Serpinb7 | Serpin peptidase inhibitor, clade B member 7 | 1.1 | 1.2 | **2.1** |
| **Apoptosis, cell cycle regulation, and oncogenesis** | | | | | |
| NM_214028 | *Inhba* | Inhibin, beta A | 1.0 | **3.4** | **2.9** |
| NM_001161640 | *Casp10* | Caspase 10 | 1.0 | **2.3** | **2.1** |
| **Complement Cascade** | | | | | |
| NM_001244102 | Vsig4 | V-set and immunoglobulin domain containing 4 | **2.2** | 0.5 | 0.8 |
| **Biological and metabolic process** | | | | | |
| NM_001204181 | *Akap2* | A kinase (PRKA) anchor protein 2 | 0.8 | 1.8 | **2.5** |
| ENSSSCT00000002555 | *Arg2* | Arginase, type II | 0.9 | **2.2** | **2.5** |
| ENSSSCT00000019676 | *Atp6* | ATP synthase F0 subunit 6 | 0.9 | **2.1** | **2.0** |
| ENSSSCT00000019675 | *Atp8* | ATP synthase F0 subunit 8 | 0.8 | **2.6** | **2.3** |
| ENSSSCT00000011170 | *Bicc1* | Bicaudal C homolog 1 | 1.5 | **2.1** | **2.4** |
| NM_214081 | *Chgb* | Chromogranin B | 1.2 | **2.1** | 1.8 |
| NM_214034 | *Chrm1* | cholinergic receptor, muscarinic 1 | 0.5 | **2.5** | 1.8 |
| NM_001044551 | *Ckmt2* | Creatine kinase, mitochondrial 2 | 0.9 | 1.0 | **2.5** |
| NM_001113015 | *Cmah* | Cytidine monophosphate-N-acetylneuraminic acid hydroxylase | 1.1 | **2.3** | **2.3** |
| XM_001928087 | *Col6a3* | Collagen, type VI, alpha 3 | 1.4 | **2.1** | 1.9 |
| ENSSSCT00000019670 | *Cox1* | Cytochrome c oxidase subunit I | 1.2 | 1.9 | **2.1** |
| ENSSSCT00000019673 | *Cox2* | Cytochrome c oxidase subunit II | 1.0 | **2.1** | **3.4** |
| ENSSSCT00000019677 | *Cox3* | Cytochrome c oxidase subunit III | 1.0 | **2.2** | **2.2** |
| NM_214414 | *Cyp2c33* | Cytochrome P450 2C33 | 1.3 | **2.1** | 1.3 |
| ENSSSCT00000019689 | *Cytb* | Cytochrome b | 1.0 | **2.1** | **2.1** |
| NM_001244160 | *Dhrs7* | Dehydrogenase/reductase (SDR family) member 7 | **2.2** | 0.8 | 1.6 |
| NM_001171750 | *Fads2* | Fatty acid desaturase 2 | 1.2 | **2.0** | **2.0** |
| AK231687 | *Fbox32* | F-box protein 32 | 0.9 | **2.5** | **2.4** |
| NM_214064 | *Fmo1* | Flavin containing monooxygenase 1 | 1.0 | **2.0** | 1.5 |
| NM_001174056 | *Gdf15* | Growth differentiation factor 15 | 0.8 | **2.1** | **2.1** |
| Z80109 | *Gem* | GTP binding protein overexpressed in skeletal muscle | 0.8 | **3.4** | **4.3** |
| NM_213822 | *Gltp* | Glycolipid transfer protein | 1.8 | 1.0 | **2.4** |
| AK237750 | *Gpr4* | G protein-coupled receptor 4 | 0.3 | **2.5** | 1.6 |
| NM_001018032 | *Gucy1b3* | Guanylate cyclase 1, soluble, beta 3 | 0.7 | **2.0** | 1.7 |
| NM_001098588 | *Hps3* | Hermansky-Pudlak syndrome 3 | 1.0 | 1.8 | **2.1** |
| NM_214028 | *Inhba* | Inhibin, beta A | 0.6 | **3.4** | **2.9** |
| NM_001245012 | *Inpp1* | Inositol polyphosphate-1-phosphatase | 1.3 | 1.6 | **2.4** |
| XM_001926559 | *Jag1* | Jagged 1 | 1.0 | 1.9 | **2.3** |
| NM_214449 | *Lhcgr* | Luteinizing hormone/choriogonadotropin receptor | 1.2 | **2.3** | 1.1 |
| NM_001008690 | *Mc1r* | Melanocortin 1 receptor | **2.1** | 1.6 | 1.1 |
| NM_001044609 | *Mchr2* | Melanin-concentrating hormone receptor 2 | 1.5 | 1.9 | **2.6** |
| ENSSSCT00000004157 | *Mcoln2* | Mucolipin 2 | 0.8 | **2.1** | 1.5 |
| NM_001244607 | *Mllt11* | Myeloid/lymphoid or mixed-lineage leukemia translocated to 11 | 1.0 | 1.6 | **2.4** |
| NM_001099941 | *Myot* | Myotilin | **2.5** | 1.7 | 1.0 |
| ENSSSCT00000019660 | *ND1* | NADH dehydrogenase subunit 1 | 1.4 | 1.8 | **2.4** |
| ENSSSCT00000019664 | *Nd2* | NADH dehydrogenase subunit 2 | 0.9 | **2.3** | **2.1** |
| ENSSSCT00000019682 | *Nd4* | NADH dehydrogenase subunit 4 | 0.8 | **2.3** | **2.2** |
| ENSSSCT00000019681 | *Nd4l* | NADH dehydrogenase subunit 4L | 0.9 | **2.4** | **2.2** |
| ENSSSCT00000019686 | *Nd5* | NADH dehydrogenase subunit 5 | 0.9 | **2.3** | **2.1** |
| NM_001123155 | *Olf42-3* | Olfactory receptor-like protein-like | **2.1** | 1.6 | 1.2 |
| NM_001161753 | *Pck2* | Phosphoenolpyruvate carboxykinase 2 | 1.0 | **2.1** | **2.3** |
| NM_001159306 | *Pdk4* | Pyruvate dehydrogenase kinase, isozyme 4 | 0.9 | 1.2 | **2.4** |
| NM_214054 | *Plat* | Plasminogen activator, tissue | 0.7 | **2.0** | **2.3** |
| NM_001244629 | *Plbd1* | Phospholipase B domain containing 1 | **2.1** | 0.6 | 0.8 |
| ENSSSCT00000002388 | *Pnp* | Purine nucleoside phosphorylase | 0.8 | **2.1** | **2.3** |
| NM_213926 | *Prl* | Prolactin | 1.6 | **2.5** | 1.1 |
| NM_001164649 | *Slc1a1* | Solute carrier family 1 member 1 | 1.1 | **2.8** | **3.5** |
| AK349467 | *Slc2a6* | Solute carrier family 2 member 6 | 1.0 | **2.0** | **2.5** |
| XM_003125519 | *Sybu* | Syntabulin | 0.8 | 1.5 | **2.2** |
| ENSSSCT00000007403 | *Syt6* | Synaptotagmin VI | 1.1 | **2.5** | **2.9** |
| ENSSSCT00000006357 | *Tarsl2* | Threonyl-tRNA synthetase-like 2 | 1.1 | 1.5 | **2.1** |
| ENSSSCT00000000799 | *Tead4* | TEA domain family member 4 | 0.9 | **2.0** | 1.7 |
| AM283538 | *Tff1* | Trefoil factor 1 | 1.5 | **2.2** | 1.7 |
| ENSSSCT00000006315 | *Vav2* | Vav 2 guanine nucleotide exchange factor | 1.2 | 1.4 | **2.1** |
