# Supplementary material for: Transcriptional Analysis of PRRSV-Infected Porcine Dendritic Cell Response to Streptococcus suis Infection Reveals Up-Regulation of Inflammatory-Related Genes Expression
Source: PLoS One. 2016 May 23;11(5):e0156019. doi: 10.1371/journal.pone.0156019 (PMC4877111; doi:10.1371/journal.pone.0156019)
Supplement: S4 Table — (DOCX) [file pone.0156019.s004.docx]

**Supplemental Table S4:** Genes downregulated greater than two-fold in porcine monocytes infected with PRRSV, *S. suis*, or co-infected with both pathogens for 12 h, compared to mock-infected cells

| **Genebank ID** | **Gene** | | **Gene description** | | **PRRSV** | ***S. suis*** | **Co-infection** |
| --- | --- | --- | --- | --- | --- | --- | --- |
| **Cytokines, chemokines, and related receptors** | | | | | | | |
| NM_001112690 | | *April* | | A proliferation-inducing ligand | 0.9 | **2.1** | **2.2** |
| NM_001001621 | | *Ccr1* | | Chemokine (C-C motif) receptor 1 | 0.8 | **2.9** | **2.4** |
| NM_001001619 | | *Ccr2* | | Chemokine (C-C motif) receptor 2 | 0.7 | **2.0** | **2.0** |
| NM_001001620 | | *Ccr3* | | Chemokine (C-C motif) receptor 3 | 1.1 | **2.8** | **3.3** |
| ENSSSCT00000017621 | | *Cxcr2* | | Chemokine (C-X-C motif) receptor 2 | 1.2 | **3.7** | **3.9** |
| NM_214135 | | *Il7* | | Interleukin 7 | **2.5** | 1.5 | **2.6** |
| NM_213751 | | *Il16* | | Interleukin 16 | 0.9 | **2.0** | **2.0** |
| NM_001204370 | | *Tnfaip8l2* | | Tumor necrosis factor, alpha-induced protein 8-like 2 | 1.0 | **2.1** | **2.0** |
| XM_003128430 | | *Tnfrsf21* | | tumor necrosis factor receptor superfamily, member 21 | 1.0 | **2.7** | **2.8** |
| **Host defense** | | | | | | | |
| AK235168 | | *Ap2m1* | | Adaptor-related protein complex 2, mu 1 subunit | 1.1 | 1.9 | **2.2** |
| ENSSSCT00000011493 | | *Blnk* | | B-cell linker | 0.9 | **2.1** | **2.3** |
| NM_001244209 | | *Calm3* | | Calmodulin 3 | 0.9 | **2.3** | **2.1** |
| ENSSSCT00000000700 | | *Clec7a* | | C-type lectin domain family 7, member A | 0.8 | **2.7** | **2.4** |
| NM_001244157 | | *Fkbp3* | | FK506 binding protein 3, 25kDa | 1.0 | **2.0** | **2.1** |
| ENSSSCT00000017252 | | *Gxr* | | Glutathione reductase | 1.0 | **2.1** | **2.3** |
| NM_001244363 | | *Ifit1* | | Interferon-induced protein with tetratricopeptide repeats 1 | 0.8 | **3.0** | **2.1** |
| NM_001244410 | | *Lcn2* | | Lipocalin 2 | 1.3 | **3.3** | **3.3** |
| NM_001123142 | | *Ncf2* | | Neutrophil cytosolic factor 2 | 1.2 | 1.7 | **2.2** |
| NM_001114277 | | *Nod1* | | Nucleotide-binding oligomerization domain containing 1 | 1.2 | 1.8 | **2.0** |
| NM_214303 | | *Oas1* | | 2'-5'-oligoadenylate synthetase 1, 40/46kDa | 0.4 | **2.9** | 1.6 |
| AK346242 | | *Ppp1r3c* | | Protein phosphatase 1, regulatory subunit 3C | 0.8 | **2.6** | **2.1** |
| NM_001244186 | | *Pstpip1* | | Proline-serine-threonine phosphatase interacting protein 1 | 1.1 | **2.2** | **2.1** |
| ENSSSCT00000008011 | | *Sla2* | | Src-like-adaptor 2 | 1.2 | **2.6** | **2.3** |
| NM_001113039 | | *Tlr4* | | Toll-like receptor 4 | 1.2 | 1.9 | **2.3** |
| AK234441 | | *Tmem110* | | Transmembrane protein 110 | 1.0 | **2.3** | **2.3** |
| NM_213756 | | *Trem1* | | Triggering receptor expressed on myeloid cells 1 | 0.9 | **2.4** | 1.8 |
| NM_001244102 | | *Vsig4* | | V-set and immunoglobulin domain containing 4 | 0.5 | **2.2** | 1.2 |
| **Surface receptor molecule and antigen presentation** | | | | | | | |
| NM_001243435 | | *Adora3* | | Adenosine A3 receptor | 1.3 | **2.6** | **3.3** |
| NM_213815 | | Cd55 | | CD55 molecule | 1.4 | **2.0** | **2.5** |
| NM_213976 | | *Cd163* | | CD163 molecule | 1.0 | **3.8** | **3.5** |
| AK239741 | | *Cd3d* | | CD3d molecule, delta | 1.2 | **3.4** | **2.7** |
| NM_001008686 | | *Cd3g* | | CD3g molecule, gamma | 1.4 | **3.6** | **4.1** |
| NM_001123112 | | *Fcar* | | Fc fragment of IgA, receptor for (CD89) | 1.0 | **2.6** | **2.3** |
| NM_001033013 | | *Fcgr2b* | | Fc fragment of IgG, low affinity IIb, receptor (CD32) | 1.2 | **2.0** | **2.3** |
| NM_214391 | | *Fcgr3b* | | Fc fragment of IgG, low affinity IIIb, receptor (CD16b) | 1.1 | **2.0** | **2.2** |
| NM_213830 | | *Folr1* | | Folate receptor 1 | 1.2 | **3.3** | **2.8** |
| AK237750 | | *Gpr4* | | G protein-coupled receptor 4 | **3.0** | 0.4 | 0.6 |
| **Cytokine signaling** | |  | |  |  |  |  |
| NM_214193 | | *Camk2g* | | Calcium/calmodulin-dependent protein kinase II gamma | 0.9 | 1.7 | **2.0** |
| NM_214320 | | *Cdkn3* | | Cyclin-dependent kinase inhibitor 3 | 1.0 | **3.1** | **2.8** |
| AK230914 | | *Irak3* | | Interleukin-1 receptor-associated kinase 3 | 1.0 | **2.2** | 1.8 |
| XM_001929490 | | *Mapk14* | | Mitogen-activated protein kinase 14 | 0.7 | 1.9 | **2.0** |
| NM_001099923 | | *Myd88* | | Myeloid differentiation primary response gene (88) | 1.3 | **2.0** | **2.4** |
| ENSSSCT00000005616 | | *Pkc* | | Protein kinase C theta | 1.0 | **2.0** | **2.2** |
| NM_214058 | | *Serpina7* | | Serpin peptidase inhibitor, clade A, member 7 | 0.9 | **2.0** | 1.4 |
| NM_001244161 | | *Serpinb10* | | Serpin peptidase inhibitor, clade B, member 10 | 1.1 | 0.8 | **2.1** |
| NM_001162889 | | *Socs4* | | Suppressor of cytokine signaling 4 | 1.3 | 1.5 | **2.0** |
| NM_213889 | | *Stat2* | | Signal transducer and activator of transcription 2 | 0.9 | **4.9** | **7.8** |
| **Cell adhesion and migration** | | | | | | | |
| ENSSSCT00000007155 | | *Adam15* | | ADAM metallopeptidase domain 15 | 1.2 | 1.8 | **2.4** |
| XM_003130751 | | *Apbb1ip* | | Amyloid beta (A4) precursor protein-binding, family B, member 1 interacting protein | 1.3 | 1.8 | **2.1** |
| AK346166 | | *Epb49* | | Erythrocyte membrane protein band 4.9 | 1.2 | **5.4** | **4.1** |
| NM_001145379 | | *Icam3* | | Intercellular adhesion molecule 3 | 1.0 | **2.3** | **2.0** |
| NM_001246669 | | *Itgb5* | | Integrin, beta 5 | 0.9 | **3.3** | **3.8** |
| ENSSSCT00000004163 | | *Lphn2* | | Latrophilin 2 | 1.0 | **2.2** | **2.7** |
| NM_213907 | | *Pecam1* | | Platelet/endothelial cell adhesion molecule | 1.1 | **2.4** | **2.9** |
| NM_214346 | | *Siglec-1* | | Sialoadhesin | 0.7 | **2.5** | **2.0** |
| NM_001244536 | | *Thbs1* | | Thrombospondin 1 | 1.1 | **4.7** | **6.6** |
| NM_001145985 | | *Timp2* | | TIMP metallopeptidase inhibitor 2 | 0.9 | **2.2** | **2.1** |
| ENSSSCT00000004618 | | *Vnn2* | | Vanin 2 | 0.9 | **2.4** | **2.1** |
| ENSSSCT00000004619 | | *Vnn3* | | Vanin 3 | 1.1 | **2.0** | **2.0** |
| **Transcriptional and translational regulation** | | | | | | | |
| NM_214375 | | *Fhl1c* | | Four and a half LIM domains 1 protein, isoform C | 0.9 | **2.0** | 1.5 |
| NM_001123113 | | *Fos* | | FBJ murine osteosarcoma viral oncogene homolog | 1.1 | 1.9 | **2.3** |
| NM_001097428 | | *Irf7* | | Interferon regulatory factor 7 | 1.1 | 1.9 | **2.2** |
| NM_001134344 | | *Klf10* | | Kruppel-like factor 10 | 1.0 | **2.1** | **2.9** |
| GQ274321 | | *Klf13* | | Kruppel-like factor 13 | 1.3 | 1.6 | **2.0** |
| NM_001129967 | | *Lef1* | | Lymphoid enhancer-binding factor 1 | 1.1 | **3.4** | **2.4** |
| NM_001005154 | | *Myc* | | V-myc | 0.9 | **3.2** | **2.9** |
| NM_001144930 | | *Nfyb* | | Nuclear transcription factor Y, beta | 1.2 | 1.6 | **2.0** |
| NM_001101814 | | *Nr1h3* | | Nuclear receptor subfamily 1, group H, member 3 | 0.9 | **2.2** | **2.2** |
| ENSSSCT00000005904 | | *Tdrd7* | | Tudor domain containing 7 | 1.1 | 1.8 | **2.2** |
| ENSSSCT00000007376 | | *Ttf2* | | Transcription termination factor, RNA polymerase II | 0.8 | **2.0** | 1.9 |
| **Vascular homeostasis, wound healing** | | | | | | | |
| NM_001005152 | | *Fgl2* | | Fibrinogen-like 2 | 1.8 | **3.4** | **5.1** |
| EW422073 | | *Hbe1* | | Hemoglobin, epsilon 1 | 1.2 | **4.9** | **4.4** |
| NM_001146130 | | *Hpse* | | Heparanase | 0.8 | **6.3** | **4.9** |
| ENSSSCT00000001103 | | *Serpinb1* | | Serpin peptidase inhibitor, member 1 | 1.0 | **3.6** | **3.4** |
| **Apoptosis, cell cycle regulation, and oncogenesis** | | | | | | | |
| ENSSSCT00000008202 | | *Aurka* | | Aurora kinase A | 1.1 | 1.8 | **2.2** |
| AK230496 | | *Casp3* | | Caspase 3 | 1.2 | **2.0** | **2.2** |
| NM_001123095 | | *Cdc25c* | | Cell division cycle 25 homolog | 1.5 | 1.7 | **2.3** |
| NM_214320 | | *Cdkn3* | | Cyclin-dependent kinase inhibitor 3 | 1.0 | **3.1** | **2.8** |
| NM_001123104 | | *Ctsz* | | Cathepsin Z | 0.7 | **2.7** | **2.2** |
| NM_001243602 | | *Id3* | | Inhibitor of DNA binding 3 | 0.7 | **5.2** | **4.3** |
| NM_001129967 | | *Lef1* | | Lymphoid enhancer-binding factor 1 | 1.1 | **3.4** | **2.4** |
| NM_001123174 | | *Rab22a* | | RAB22A, member RAS oncogene family | 1.1 | 1.7 | **2.1** |
| XM_001928649 | | *Rap1a* | | RAP1A, member of RAS oncogene family | 1.2 | 1.6 | **2.1** |
| NM_001129960 | | *Rtn2* | | Reticulon 2 | 1.0 | **3.4** | **3.3** |
| NM_001044614 | | *Txnip* | | Thioredoxin interacting protein | 1.1 | **5.8** | **6.7** |
| **Complement cascade** | | | | | | | |
| NM_001244215 | | *C5ar1* | | Complement component 5a receptor 1 | 1.2 | **2.7** | **3.6** |
| NM_214281 | | *Cfh* | | Complement factor H | 0.8 | **3.1** | 1.8 |
| NM_214160 | | *Fcn1* | | Ficolin 1 | 0.7 | **3.5** | **2.5** |
| **Lipid Metabolism** | | | | | | | |
| NM_001143715 | | *Lrp10* | | Low density lipoprotein receptor-related protein 10 | 1.3 | 1.6 | **2.1** |
| NM_213739 | | *Paqr7* | | Progestin and adipoQ receptor family member VII | 1.1 | **2.3** | **2.3** |
| AK236973 | | *Plcl2* | | Phospholipase C-like 2 | 0.9 | **2.1** | **2.0** |
| NM_001143722 | | *Pitpnc1* | | Phosphatidylinositol transfer protein, cytoplasmic 1 | 0.7 | **2.2** | 1.7 |
| NM_001244155 | | *Scarb2* | | Scavenger receptor class B, member 2 | 1.0 | **2.1** | **2.5** |
| ENSSSCT00000011246 | | *Sgpl1* | | Sphingosine-1-phosphate lyase 1 | 1.2 | 1.8 | **2.3** |
| **Biological and metabolic process** | | | | | | | |
| NM_214039 | | *Acadm* | | Acyl-CoA dehydrogenase | 0.9 | 1.9 | **2.0** |
| NM_001206399 | | *Acacb* | | Acetyl-CoA carboxylase beta | 0.9 | **2.1** | 1.6 |
| NM_001143695 | | *Acss2* | | Acyl-CoA synthetase short-chain family member 2 | 0.8 | **2.1** | 1.8 |
| NM_214226 | | *Adap1* | | ArfGAP with dual PH domains 1 | 1.0 | **2.3** | 1.6 |
| NM_001243435 | | *Adora3* | | Adenosine A3 receptor | 1.0 | **2.8** | **2.7** |
| XM_003129347 | | *Agpat9* | | 1-acylglycerol-3-phosphate O-acyltransferase 9 | 1.0 | **2.0** | **2.0** |
| NM_001044611 | | *Aldh2* | | Aldehyde dehydrogenase 2 family | 0.8 | **3.2** | **3.0** |
| NM_001206449 | | *Ankrd28* | | Ankyrin repeat domain 28 | 1.2 | 1.5 | **2.1** |
| ENSSSCT00000012366 | | *Ano10* | | Anoctamin 10 | 0.9 | **2.0** | **2.1** |
| NM_001190239 | | *Arpc2* | | Actin related protein 2/3 complex, subunit 2, 34kDa | 1.1 | **2.1** | **2.2** |
| ENSSSCT00000012245 | | *Aste1* | | Asteroid homolog 1 | 0.8 | 1.9 | **2.0** |
| AY609491 | | *Atp1b1* | | ATPase, Na+/K+ transporting, beta 1 polypeptide | 1.0 | **2.1** | 1.8 |
| X15073 | | *Atp2a2* | | ATPase, Ca++ transporting, cardiac muscle, slow twitch 2 | 1.0 | **2.2** | **2.1** |
| NM_001114672 | | *Atp9a* | | ATPase, class II, type 9A | 0.7 | **2.1** | 1.6 |
| AK238030 | | *Aqp1* | | Aquaporin 1 | 1.2 | **5.1** | **4.2** |
| NM_214351 | | *B3galnt1* | | Beta-1,3-N-acetylgalactosaminyltransferase 1 | 1.2 | **3.6** | **2.6** |
| AK239727 | | *B4galt1* | | UDP-Gal:betaGlcNAc beta 1,4- galactosyltransferase, polypeptide 1 | 1.1 | 1.8 | **2.2** |
| NM_214171 | | *Capn3* | | Calpain 3 | 0.7 | **2.3** | **2.2** |
| NM_001097456 | | *Capzb* | | Capping protein (actin filament) muscle Z-line, beta | 0.9 | **2.1** | 1.0 |
| M20160 | | *Cast* | | Calpastatin | 1.1 | **2.1** | **2.2** |
| NM_214301 | | *Cat* | | Catalase | 0.6 | **2.2** | 1.4 |
| NM_001044534 | | *Cds1* | | CDP-diacylglycerol synthase 1 | 0.8 | **2.3** | **2.1** |
| NM_214016 | | *Cecr1* | | Cat eye syndrome chromosome region, candidate 1 | 0.8 | **2.9** | **2.7** |
| AK343509 | | *Clstn1* | | Calsyntenin 1 | 0.8 | **2.2** | **2.1** |
| NM_213971 | | *Clu* | | Clusterin | 1.3 | 1.7 | **2.0** |
| NM_001097500 | | *Cox8h* | | COX8H protein | 1.3 | **2.0** | 1.3 |
| NM_214367 | | *Ctnnb1* | | Catenin beta 1 | 1.3 | 1.8 | **2.2** |
| AK240289 | | *Cttnbp2nl* | | CTTNBP2 N-terminal like | 1.2 | 1.9 | **2.1** |
| NM_001001770 | | *Cyb5a* | | Cytochrome b5 type A (microsomal) | 0.7 | **2.1** | 1.6 |
| NM_001128452 | | *Cybrd1* | | Cytochrome b reductase 1 | 1.1 | 1.8 | **2.0** |
| NM_214412 | | *Cyp1a1* | | Cytochrome P450 1A1 | 1.3 | **3.6** | **3.6** |
| NM_001195114 | | *Dybll1* | | Dynein, light chain, LC8-type 1 | 1.1 | 1.9 | **2.2** |
| NM_001044607 | | *Ech1* | | Enoyl CoA hydratase 1, peroxisomal | 0.8 | **2.1** | 1.7 |
| NM_001044527 | | *Eno3* | | Enolase 3 | 0.8 | **3.3** | **2.2** |
| NM_214355 | | *Ephx1* | | Epoxide hydrolase 1, microsomal (xenobiotic) | 0.9 | 1.7 | **2.3** |
| NM_001143706 | | *Erlin2* | | ER lipid raft associated 2 | 0.9 | 1.9 | **2.5** |
| XM_003127993 | | *Eps15* | | Epidermal growth factor receptor pathway substrate 15 | 0.9 | **2.4** | **2.0** |
| NM_001244372 | | *F2R* | | coagulation factor II (thrombin) receptor | 0.8 | **2.4** | **2.1** |
| ENSSSCT00000011681 | | *Fam45a* | | Family with sequence similarity 45, member A | 1.3 | 1.8 | **2.2** |
| AK240364 | | *Flna* | | Filamin A, alpha | 1.1 | 1.6 | **2.1** |
| NM_213830 | | *Folr1* | | Folate receptor 1 | 0.8 | **3.2** | **2.6** |
| NM_214068 | | *Fut1* | | Fucosyltransferase 1 | 1.2 | **2.1** | 1.1 |
| ENSSSCT00000001660 | | *Gclc* | | Glutamate-cysteine ligase, catalytic subunit | 0.9 | **2.5** | **2.6** |
| NM_214030 | | *Ggt1* | | Gamma-glutamyltransferase 1 | 0.8 | **2.1** | 1.9 |
| ENSSSCT00000007560 | | *Glmn* | | Glomulin | 1.0 | **2.6** | **2.9** |
| AK237209 | | *Glo1* | | Glyoxalase I | 0.8 | **2.0** | 1.6 |
| NM_001244501 | | *Glud1* | | Glutamate dehydrogenase 1 | 0.9 | **2.0** | 1.8 |
| NM_001243376 | | *Gnpnat1* | | Glucosamine-phosphate N-acetyltransferase 1 | 1.2 | **2.0** | **2.0** |
| NM_001243638 | | *Gstz1* | | Glutathione transferase zeta 1 | 0.9 | **2.3** | **2.1** |
| NM_214299 | | *Hbegf* | | Heparin-binding EGF-like growth factor | 1.0 | **2.0** | 1.6 |
| NM_001004027 | | *Hmox1* | | Heme oxygenase 1 | 1.2 | **2.2** | **2.6** |
| NM_001123043 | | *Hpcal4* | | Hippocalcin like 4 | 1.2 | 1.4 | **2.0** |
| NM_001098587 | | *Hps1* | | Hermansky-Pudlak syndrome 1 | 0.8 | **2.2** | 1.9 |
| NM_214248 | | *Hsd11b1* | | Hydroxysteroid (11-beta) dehydrogenase 1 | 0.8 | **3.5** | **2.3** |
| NM_001042375 | | *Itm2b* | | Integral membrane protein 2B | 0.8 | **2.6** | **2.1** |
| NM_214093 | | *Kcne3* | | Potassium voltage-gated channel, Isk-related family, member 3 | 1.0 | 1.8 | **2.0** |
| ENSSSCT00000000542 | | *Kcnmb4* | | Potassium large conductance calcium-activated channel, subfamily M, beta member 4 | 1.0 | **2.0** | **2.4** |
| ENSSSCT00000001683 | | *Kifc1* | | Kinesin family member C1 | 1.2 | 1.8 | **2.1** |
| ENSSSCT00000010329 | | *Lacc1* | | Laccase domain containing 1 | 1.0 | 1.8 | **2.0** |
| NM_001243410 | | *Lactb2* | | Lactamase, beta 2 | 0.9 | **2.1** | **2.0** |
| NM_001111257 | | *Lmna* | | Lamin A/C | 1.3 | 1.6 | **2.2** |
| NM_001145388 | | *Leprot* | | Leptin receptor overlapping transcript | 1.2 | **2.0** | **2.5** |
| ENSSSCT00000004721 | | *Lmbrd1* | | LMBR1 domain containing 1 | 1.1 | 1.7 | **2.2** |
| AK231743 | | *Mthfr* | | Methylenetetrahydrofolate reductase (NAD(P)H) | 1.1 | 1.9 | **2.4** |
| AK235300 | | *Mylk* | | Myosin light chain kinase | 1.1 | **2.3** | **2.7** |
| NM_001243484 | | *Nceh1* | | Neutral cholesterol ester hydrolase 1 | 0.7 | **4.0** | **3.3** |
| NM_214071 | | *Npl* | | N-acetylneuraminate pyruvate lyase | 1.0 | **2.7** | **3.0** |
| NM_214288 | | *Npy1r* | | Neuropeptide Y receptor Y1 | 1.2 | 1.8 | **2.2** |
| NM_001159613 | | *Nqo1* | | NAD(P)H dehydrogenase, quinone 1 | 1.0 | **2.5** | **2.3** |
| AY609888 | | *Nsdhl* | | NAD(P) dependent steroid dehydrogenase-like | 1.0 | **2.2** | **2.6** |
| ENSSSCT00000010538 | | *Nudt18* | | Nudix-type motif 18 | 1.1 | **2.1** | 1.8 |
| NM_001243685 | | *Obfc1* | | Pligonucleotide/oligosaccharide-binding fold containing 1 | 1.1 | 1.9 | **2.7** |
| ENSSSCT00000013264 | | *Ofd1* | | Oral-facial-digital syndrome 1 | 1.1 | 1.9 | **2.1** |
| NM_214005 | | *Ostf1* | | Osteoclast stimulating factor 1 | 1.0 | **2.0** | 1.9 |
| ENSSSCT00000012831 | | *P2ry1* | | Purinergic receptor P2Y, G-protein coupled, 1 | 0.8 | **2.9** | **2.4** |
| ENSSSCT00000003774 | | *Pgd* | | Phosphogluconate dehydrogenase | 0.8 | **2.1** | **2.0** |
| XM_001926780 | | *Plekho2* | | Pleckstrin homology domain containing, family O member 2 | 1.2 | 1.9 | **2.3** |
| NM_001168416 | | *Pmvk* | | Phosphomevalonate kinase | 1.1 | **2.3** | **2.5** |
| XM_003121446 | | *Poli* | | Polymerase (DNA directed) iota | 1.0 | 1.9 | **2.1** |
| AK234922 | | *Pon2* | | Paraoxonase 2 | 0.9 | **2.1** | **2.0** |
| NM_001244164 | | *Prim2* | | Primase, DNA, polypeptide 2 | 0.9 | **2.5** | **2.1** |
| NM_001129963 | | *RTN4* | | Reticulon 4 | 1.2 | 1.6 | **2.1** |
| ENSSSCT00000012352 | | *Sec22c* | | SEC22 vesicle trafficking protein homolog C | 1.2 | **2.4** | 1.6 |
| EF113595 | | *Sepn1* | | Selenoprotein N, 1 | 1.0 | 1.8 | **2.2** |
| NM_001244148 | | *Serinc2* | | Serine incorporator 2 | 1.1 | **2.5** | **2.6** |
| ENSSSCT00000006231 | | *Sh3glb2* | | SH3-domain GRB2-like endophilin B2 | 1.0 | 1.9 | **2.1** |
| NM_001114270 | | *Sirt4* | | Sirtuin 4 | 0.8 | **2.3** | 1.7 |
| NM_001143724 | | *Slc9a3r1* | | Solute carrier family 9 member 3 regulator 1 | 1.1 | **2.7** | **2.8** |
| NM_001243383 | | *Slc46a1* | | Solute carrier family 46, member 1 | 0.8 | **2.2** | 1.9 |
| AK233023 | | *Slc46a3* | | Solute carrier family 46, member 3 | 0.8 | **2.0** | 1.4 |
| NM_001244216 | | *Snx6* | | Sorting nexin 6 | 1.2 | **2.0** | **2.2** |
| NM_213864 | | *Spink4* | | Serine peptidase inhibitor, Kazal type 4 | 1.3 | **9.2** | **5.6** |
| NM_214238 | | *Sprp* | | Small proline-rich protein | 1.5 | 1.7 | **2.1** |
| NM_213765 | | *Sult1a1* | | Sulfotransferase family, cytosolic, 1A, phenol-preferring, member 1 | 0.8 | **2.0** | 1.8 |
| ENSSSCT00000012909 | | *Tbccd1* | | TBCC domain containing 1 | 1.0 | **2.4** | **2.4** |
| XM_001926631 | | *Tbc1d12* | | TBC1 domain family, member 12 | 1.2 | **2.1** | **2.1** |
| AK230950 | | *Tcn1* | | Transcobalamin I | 1.3 | **3.9** | **4.3** |
| ENSSSCT00000004332 | | *Tesk2* | | Testis-specific kinase 2 | 1.0 | **2.3** | **2.0** |
| NM_001112681 | | *Tkt* | | Transketolase | 0.9 | **2.3** | **2.2** |
| NM_213912 | | *Tnni1* | | troponin I type 1 | 1.3 | 1.5 | **2.4** |
| NM_001097483 | | *Tpm1* | | Tropomyosin 1 (alpha) | 0.7 | **2.2** | **2.1** |
| NM_001243490 | | *Tpst2* | | Tyrosylprotein sulfotransferase 2 | 0.9 | **2.1** | **2.2** |
| ENSSSCT00000011229 | | *Tysnd1* | | Trypsin domain containing 1 | 0.9 | 1.8 | **2.1** |
| NM_214104 | | *Vtn* | | Vitronectin | 1.0 | **2.1** | **2.2** |
